# Altitudinal and seasonal distribution of butterflies (Lepidoptera, Papilionoidea) in Cerro Bufa El Diente, Tamaulipas, Mexico

**DOI:** 10.3897/zookeys900.36978

**Published:** 2019-12-31

**Authors:** Edmar Meléndez-Jaramillo, César Cantú-Ayala, Uriel Jeshua Sánchez-Reyes, Fatima Magdalena Sandoval-Becerra, Bernal Herrera-Fernández

**Affiliations:** 1 Facultad de Ciencias Forestales, Universidad Autónoma de Nuevo León, Ap. Postal 41, Linares, Nuevo León, C. P. 67700, México Universidad Autónoma de Nuevo León Linares Mexico; 2 Tecnológico Nacional de México - Instituto Tecnológico de Cd. Victoria. Boulevard Emilio Portes Gil No.1301, C.P. 87010. Ciudad Victoria, Tamaulipas, México Instituto Tecnológico de Ciudad Victoria Victoria Mexico; 3 Fundación para el Desarrollo de la Cordillera Volcánica Central (Fundecor), Costa Rica e Instituto Internacional para la Conservación y Manejo de la Vida Silvestre (Icomvis), Universidad Nacional, Heredia, Costa Rica Universidad Nacional Heredia San José Costa Rica

**Keywords:** Diurnal Lepidoptera, diversity, elevation, indicator species, priority land region, seasonality

## Abstract

Butterflies are one of the most recognized and useful groups for the monitoring and establishment of important conservation areas and management policies. In the present study, we estimate the richness and diversity, as well as the association value of submontane scrub, oak forest, and cloud forest species at Cerro Bufa El Diente, within the Sierra de San Carlos priority land region, located in the Central-western region of Tamaulipas, Mexico. Three sampling sites were established based on criteria of vegetation distribution per altitudinal floor. One site for each altitudinal floor and vegetation type. Sampling was carried out in permanent transects on a monthly basis at each site, using an aerial entomological net and ten Van Someren-Rydon traps, during four sampling periods: early dry season, late dry season, early wet season and late wet season. In total, 7,611 specimens belonging to six families, 20 subfamilies, 32 tribes, 148 genera and 243 species of the study area were collected. Nymphalidae was the most abundant family with 3,454 specimens, representing 45.38% of total abundance in the study area. Lower abundance was recorded in Hesperiidae (19.17%), Pieridae (16.41%), Lycaenidae (10.17%), Papilionidae (5.12%), and finally Riodinidae (3.74%). The highest species richness was presented in the family Hesperiidae with 34.57% of the total obtained species followed by Nymphalidae (30.45%), Lycaenidae (15.23%), Pieridae (9.88%), Papilionidae (5.76%), and Riodinidae (4.12%). Twenty-seven species were categorized as abundant, these species, *Anaea aidea* (Guérin-Méneville, 1844), *Libytheana carinenta larvata* (Strecker, 1878), *Pyrgus oileus* (Linnaeus, 1767), *Mestra amymone* (Ménétriés, 1857) and *Phoebis agarithe agarithe* (Boisduval, 1836) presented the highest number of specimens. Sixty-five species were considered common, constituting 41.73% of the total number of butterflies, 63 frequent (9.76% of the total abundance), 55 limited (2.54%) and 33 rare (0.43%). The greatest number of specimens and species, as well as alpha diversity, were presented on the lowest altitudinal floor, made up of submontane scrub, and decreased significantly with increasing altitude. According to the cluster analysis, low and intermediate altitude sites constitute an area of distribution of species that prefer tropical conditions, while the third-floor site forms an independent group of high mountain species. The greatest abundance and richness of species, as well as alpha diversity, was obtained during the last wet season, decreasing significantly towards the early dry season. Moreover, through the use of the association value, 19 species were designated as indicators, three for the last altitudinal floor, three for the intermediate and 13 for the first. The present work represents the first report of the altitudinal variation in richness, abundance and diversity of butterflies in the northeast of Mexico. These results highlight the importance of the conservation of this heterogeneous habitat and establish reference data for the diurnal Lepidoptera fauna of the region.

## Introduction

More than 155,000 species of Lepidoptera have been described to date (Nieukerken et al. 2011), as such the order comprise 10% of the known animal diversity (Kristensen et al. 2007). The Butterflies (Papilionoidea) comprise six families: Papilionidae, Pieridae, Lycaenidae, Riodinidae, Nymphalidae and Hesperiidae, and together represent 13% of total species in Lepidoptera worldwide (Kawahara and Breinholt 2014; Llorente et al. 2014). In Mexico, according to Warren (2000), Llorente et al. (2006) and Llorente et al. (2014), it is estimated that there are 2,049 species, corresponding to 9.4% of the Papilionoidea described worldwide. Butterflies are among the best environment quality indicator insects, because they are highly diverse and abundant (Prince-Chacón et al. 2011), easy to identify at field and due to their rapic biological cycles, they are easy to sample in any time of the year (Freitas et al. 2006). In addition, they are affected by constant landscape changes, because they are closely related to the vegetation (Marín et al. 2014), and most of their life cycle is associated with specific plants (Orozco et al. 2009). Furthermore, they respond to the stratification of the vegetation in terms of light, wind, humidity and temperature gradients (Montero-Muñoz et al. 2013). Therefore, they are very sensitive to climatic and ecological variations occurring in natural gradients, such as elevation (Camero et al. 2007).

Numerous studies show the close association between altitude and changes in composition and diversity of species (Muñoz and Amarillo-Suárez 2010). Several hypotheses have been proposed to explain among which the Rapoport effect states that the richness and distribution ranges of species are inversely related to altitude, with higher richness at low elevations (Sanders 2002), while the hypothesis of average domain indicates that the greatest number of species occurs at intermediate altitudes (Brown and Lomolino 1998). Besides, McCoy (1990) determined that, if the distribution differs between elevations, then the time scale used would strongly influence the evaluation of species richness. Thus, seasonal variations are strongly linked to elevational patterns of communities (Castro and Espinosa 2016).

In Mexico, several checklists of butterflies from altitudinal transects ranging from 600 to 3,100 m asl, including different vegetation types, have been published (Llorente et al. 1986; Luis and Llorente 1990; Luis et al. 1991; Vargas et al. 1994, 1999; Díaz-Batres et al. 2001; Luna and Llorente 2004; Luna et al. 2008; Luna et al. 2010; Álvarez et al. 2016). In addition, at a temporal level, the climatic factors influencing butterfly species turnover have been addressed in previous studies (Luis and Llorente 1990, 1991; Vargas et al. 1994; Hernández-Mejía et al. 2008; Luna et al. 2008; Pozo et al. 2008; Luna et al. 2010). However, little is known about the entomofauna and especially about the butterfly ecology of the extreme, humid and dry environments of northeastern Mexico, which is inhabited by a very special group that represents about 15% of national entomofauna, and harbors elements of the Atlantic District of the United States (Luz and Madero 2011). Knowing the distribution of the species richness and abundance of butterflies in altitudinal gradients, allows to elucidate patterns and processes of biological diversification, occupying an important role to demonstrate the conservation value of a particular habitat (DeVries and Walla 2001). Likewise, the study of communities and populations of butterflies over time, can offer important information to implement urgent measures before the effects of environmental disturbance become irreversible (Núñez-Bustos et al. 2011).

In this context, the Bufa El Diente mountain constitutes one of the highest elevation gradients (up to 1,460 m asl) in the Sierra de San Carlos, which is an isolated orographic unit within the coastal plain of the North Gulf of Mexico (Treviño et al. 2002). The region is considered an area of special interest for conservation and requires an evaluation of its natural resources (Arriaga et al. 2000). The objectives of the present study were: 1) to determine the butterflies species richness in Cerro Bufa El Diente, Tamaulipas, Mexico; 2) analyze the variation of Rhopalocera species richness, abundance and diversity along an altitudinal gradient, and during different seasons of the year; 3) analyze the influence of climatological variables (temperature, precipitation, relative humidity and solar radiation) on the abundance and richness of butterfly species; and 4) quantify the indicator value of species by each altitudinal site.

## Methods

### Study area

**Figure 1.**
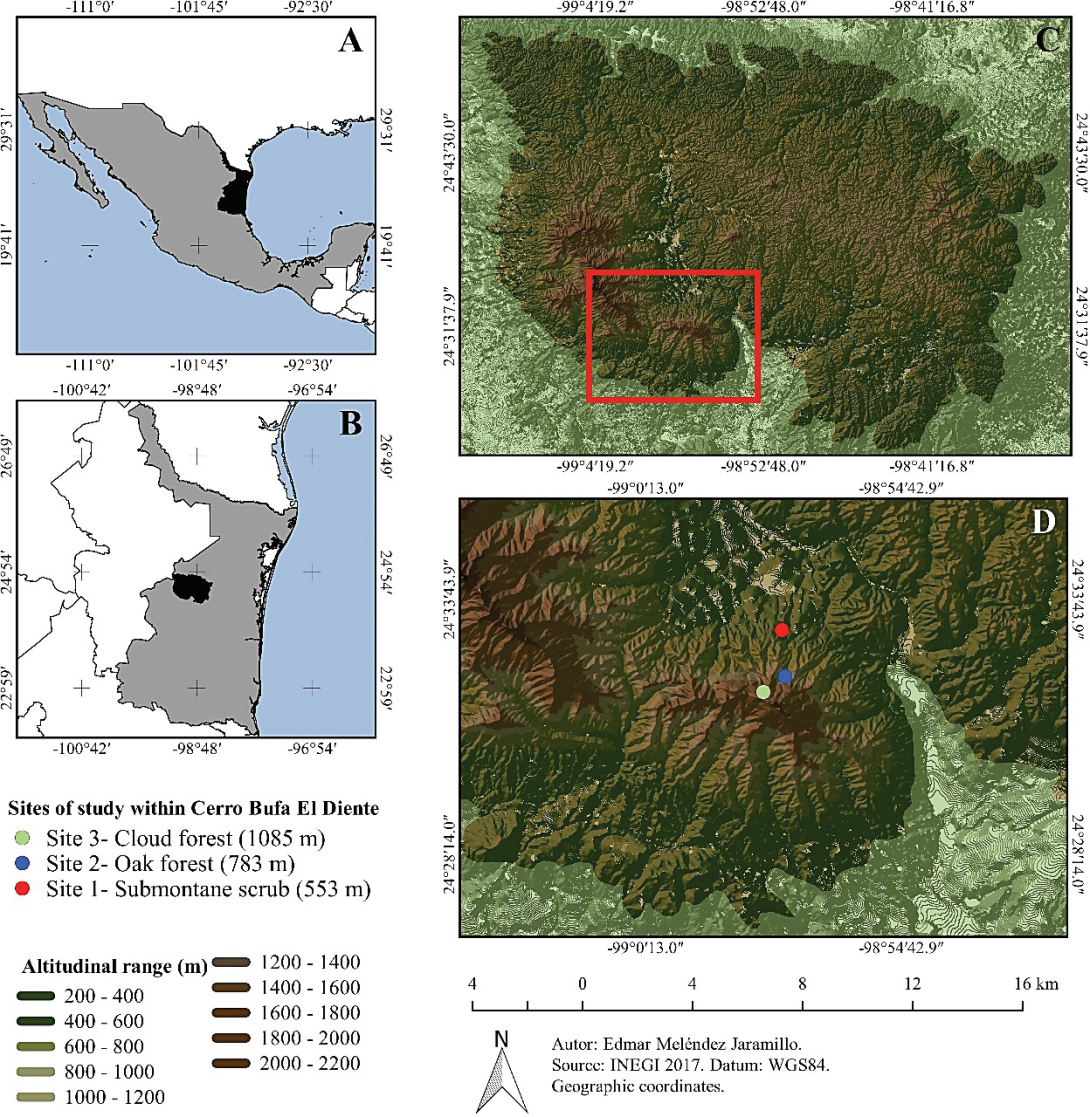
Study area and location of sampling sites **A** location of Tamaulipas in Mexico **B** location of Sierra de San Carlos within Tamaulipas **C** study area (red square) within Sierra de San Carlos **D** elevation sites in Cerro Bufa El Diente.

The Cerro Bufa El Diente mountain is located in the Sierra de San Carlos, located in the central-western portion of the State of Tamaulipas, between 24°23.03' and 24°51.60'N, and 98°32.40' and 99°12.04'W (Figure 1). Sierra de San Carlos (also known as Sierra Chiquita or Sierra de Cruillas) is a physiographic discontinuity in the Coastal Plain of the Gulf of Mexico. Due to its relative geographical isolation in relation to the Sierra Madre Oriental, it can be conceived as an ecological island, where relatively particular populations and communities have been originated or conserved (Briones-Villarreal 1991). The area is considered as a Mexican Priority Region for Conservation (RTP) of biodiversity by the National Commission for the Knowledge and Use of Biodiversity (CONABIO). The vegetation types of this RTP mainly comprise temperate ecosystems in the mountain part and submontane scrub in the piedmont (Arriaga et al. 2000). A main characteristic of the region is that it represents the boreal limit of the cloud forest in northeastern Mexico (Valdez-Tamez et al. 2003). Climate of the region is semi-warm sub-humid with summer rains; average annual temperature is 18 to 22 °C, and the annual precipitation ranges between 500 and 2,500 mm (Treviño et al. 2002).

### Site locations

Three sites were established based on Llorente (1984) and Briones (1991) criteria for the altitudinal gradient and vegetation types. Site 1 has the lowest elevation at 553 m asl and corresponds to submontane scrub (SS) (24°33.04'N, 98°57.16'W). Site 2 is located at an intermediate altitude of 783 m asl where the plant community consists of oak forest (OF) (24°32.04'N, 98°57.13'W). Site 3 is the highest elevation with 1085 m asl and a community of cloud forest (CF) (24°31.44'N, 98°57.41'W; Table 1).

**Table 1.** Synthesis of the collection sites.

**Site**	**Vegetation**	**Frequent species**	**General description**
1	Submontane scrub (SS)	The dominant shrubs are *Helietta parvifolia*, *Leucophyllum frutescens* and *Acacia rigidula*, or *Havardia pallens*, *Cordia boissieri* and *Acacia berlandieri*.	It grows in the piedmont and hillsides with south exposure, at altitudes of 500 to 800 m asl.
2	Oak forest (OF)	Along with *Quercus canbyi*, it is common to find *Arbutus xalapensis*, *Quercus clivicola* and *Quercus virginiana*, or, in addition to *Quercus rysophylla*, there are other oak species: *Q. sartorii*, *Q. laceyi*, *Q. clivicola*, as well as *Arbutus xalapensis*, *Pinus pseudostrobus*, *Persea podadenia* , *Carya ovata*, *Prunus serotina* and *Platanus occidentalis*.	It is possible to recognize two variants of this type of vegetation. The first one is the *Quercus canbyi* forest. It is found around 700 m asl on slopes with north exposure, bordering the submontane scrub. From there it extends up to 1,000 m asl, where it comes into contact with the *Quercus rysophylla* forest, which is the second variant.
3	Cloud forest (CF)	*Abies guatemalensis*, is the most abundant species, followed by *Carya ovata*. Oaks as a whole are also important, followed by *Carpinus caroliniana*, *Ostrya virginiana*, *Gleditsia triacanthos*, *Persea podadenia*, *Ilex rubra*, *Acer saccharum*, *Ungnadia speciosa* and *Crataegus rosei*.	Restricted to the upper parts of Cerro Bufa El Diente, with north exposure, between 1,300 and 1,400 m asl.

### Collection and processing of specimens

The collection of individuals was conducted using aerial entomological nets. At each site, routes were made along a 1 km permanent transect, following the techniques recommended by Villarreal et al. (2006). Also, along with the use of the aerial entomological nets, the sampling was carried out using Van Someren-Rydon traps (Rydon 1964). Ten traps were placed along a permanent transect 500 m long, at a distance of 50 m from one another, and between 1 to 2.5 m high from the ground. Bait used for the traps consisted of a fermented mixture of seasonal fruits: plantain (*Musa paradisiaca*), pineapple (*Ananas comosus*), mango (*Mangifera indica*), and guava (*Psidium guajava*).

Monthly samplings were made for each of the sites, during the period from September 2012 to August 2013, resulting in a total of three samples-months per season: Early dry season (EDS: December, January, February), Late dry season (LDS: March, April, May), Early rainy season (ERS: June, July, August), and Late rainy season (LRS: September, October, November). Seasons were defined on basis of historical data of total monthly values of temperature and precipitation (average of 1990 to 2010), which were obtained from a meteorological station located within the study area in the municipality of San Carlos. Therefore, a total of 36 sampling units (three samplings per four seasons per three sites) were considered. Additionally, for each site and date of collection, the temperature and relative humidity variables were recorded using a Kestrel 3500 portable weather station, while values of precipitation and solar radiation were extracted with QGIS 2.18 software (Quantum GIS 2017) from the WorldClim database available in http://worldclim.org/ and described by Fick and Hijmans (2017).

The collected entomological specimens were mounted according to the procedure described by Andrade et al. (2013). All specimens were labeled and deposited in the entomological collection of the Instituto Tecnológico de Cd. Victoria, Ciudad Victoria, Tamaulipas, Mexico, and in the collection of the Department of Conservation of the Faculty of Forestry Sciences at the Universidad Autónoma de Nuevo León, Linares, Nuevo León, Mexico. For taxonomic identification of specimens, the works of Scott (1986), Llorente et al. (1997), Luis et al. (2003), Garwood and Lehman (2005), Glassberg (2007), Vargas et al. (2008) and Luis et al. (2010), were consulted. Phylogenetic arrangement of species followed Warren et al. (2012).

### Data analysis

The abundance was quantified based on the total number of individuals per species collected at each site, season and for the entire study area. Five categories of species were considered according to the total abundance recorded: rare (species with one specimen), scarce (from 2 to 5), frequent (from 6 to 21), common (from 22 to 81), and abundant (with 82 or more specimens) (Luna et al. 2010). To corroborate significant differences between the abundance associated to each site, as well as to each season of the year, nonparametric tests of Kruskal-Wallis and Mann-Whitney were carried out. As a measure of specific richness, the total number of species obtained was used for each site, season and for the entire study area. A permutation test was conducted to determine significant variations in the number of species. Both tests (for abundance and species richness) were carried out using the Rcommander package (Fox 2005) in the program R 3.2.3 (R Development Core Team 2015). To calculate the potential number of species, the nonparametric estimators of Chao 1 and Jackknife 1 were used. These indices were chosen according to: 1) a distribution model of abundance is not previously assumed, 2) they are robust in calculating the minimum estimate of specific richness, 3) they are necessary as a complementary measure in biodiversity analyzes, and 4) Chao 1 considers the association between the number of species represented by an individual (singletons) and those represented by two individuals (doubletons) in the sample, while Jackknife 1 is a conservative index based on incidence data (presence or absence) of those species found only in one sample (uniques) (Magurran 2004; Hortal et al. 2006; Villarreal et al. 2006; Gotelli and Colwell 2011). The estimators were calculated with 100 randomizations without replacement using the software EstimateS 9.1 (Colwell 2013), based on the abundance of the species recorded by each sampling unit, and were obtained for each site, station of the year and for the entire study area. To complement the estimation of richness, and as a measure for the analysis of sampling efficiency, the linear dependence model was used. It assumes that as the list of species increases, the probability of adding new taxa decreases exponentially, and is an ideal model for studying small areas and known taxa (Gómez-Anaya et al. 2014). The value obtained from the coefficient of determination (R^2^) was used, as well as the slope value, which allows to measure the quality of the faunistic inventory. The calculation was based on the number of samples for each site, as well as for each season of the year and for the entire study area; the procedure was performed in the program Statistica 13.3 (TIBCO Software Inc. 2017).

In this study, alpha diversity was considered a measure of association or relation between abundance and number of species. Therefore, Simpson’s dominance index and Shannon’s entropy or uncertainty index were used for its measurement; these indices were calculated for the entire study area, as well as for each site and season using the vegan package (Oksanen et al. 2012) of the platform R 3.2.3. The SHE analysis S (species richness), H (Shannon-Wiener diversity index) and E (evenness as measured using the Shannon-Wiener evenness index) is a method that consists of analyzing the behavior of three components: diversity, the natural logarithm of evenness and the proportion of the previous two as a function of abundance (Buzas and Hayek 1996). To discriminate between the types of distribution, the component with the least variation was identified in relation to different values of number of species and abundance. If the diversity parameter remains more stable, then the distribution corresponds to a logarithmic series; if the most stable is the proportion between natural logarithm of evenness and diversity, a normal log distribution is attributed; and if evenness is the most stable, then the distribution will be of a broken stick type (Carreño-Rocabado 2006). The SHE test was carried out for the entire study area, as well as for each site using the forams package (Aluizio 2015) in R 3.2.3. Beta diversity was measured as the faunal similarity between sites and seasons, using the Bray-Curtis similarity index. In addition, a cluster analysis was carried out to define groups of sites and seasons according to their species composition, using the adjusted Euclidean units as distance measure and the Ward method as an amalgamation algorithm. Calculations were made in the Rcommander package (Fox 2005) in the R 3.2.3 program. A Spearman correlation test was applied between the monthly averages of microclimate variables (temperature, precipitation, relative humidity and solar radiation) and ecological parameters (number of species and abundance) using the Rcommander package (Fox 2005) in R 3.2.3.

Finally, to calculate the association value of each butterfly species to the habitat type, the indicator value index (IndVal) was used (Dufrene and Legendre 1997). This is based on the degree of specificity (exclusivity of the species to a particular site based on its abundance), and the degree of fidelity (frequency of occurrence within the same habitat) (Tejeda-Cruz et al. 2008), expressed in a percentage value. The analyzes were carried out in the abdsv package in platform R 3.2.3, using 1,000 random permutations to define the level of significance. Indicator species with an index equal to or greater than 75% were categorized as “characteristics”, which are defined by their high specificity to a given habitat, while species with a value less than 75% but equal to or greater than 50% considered as “detectors”, which present different degrees of preference for diverse habitats (McGeoch et al. 2002).

## Results

### Abundance, richness, and diversity of butterflies in Cerro Bufa El Diente

A total of 7,611 specimens of Papilionoidea was collected from 36 samples, between September 2012 to August 2013. These belong to six families, 20 subfamilies, 32 tribes, 148 genera, and 243 species (Appendix 1). Nymphalidae was the most abundant family with 3,454 specimens, representing 45.38% of total abundance in the study area. Lower abundance was recorded in Hesperiidae (19.17%), Pieridae (16.41%), Lycaenidae (10.17%), Papilionidae (5.12%), and finally Riodinidae (3.74%). The highest species richness was also presented found in the family Hesperiidae with 34.57% of the total obtained species followed by Nymphalidae (30.45%), Lycaenidae (15.23%), Pieridae (9.88%), Papilionidae (5.76%), and Riodinidae (4.12%). Twenty-seven species were categorized as abundant (with more than 82 specimens) and accounted for 45.54% of the total abundance. These abundant species, *Anaea aidea* (Guérin-Méneville, 1844) (442 individuals), *Libytheana carinenta larvata* (Strecker, 1878) (213), *Pyrgus oileus* (Linnaeus, 1767) (176), *Mestra amymone* (Ménétriés, 1857) (172) and *Phoebis agarithe agarithe* (Boisduval, 1836) (167), among others presented the highest number of specimens. Sixty-five species were considered common, constituting 41.73% of the total number of butterflies. Sixty-three species were considered frequent (743 specimens) by occupying 9.76% of the total abundance. Fifty-five species were scarce (2.54% of total abundance) and 33 were rare (0.43%) (Appendix 1).

**Figure 2.**
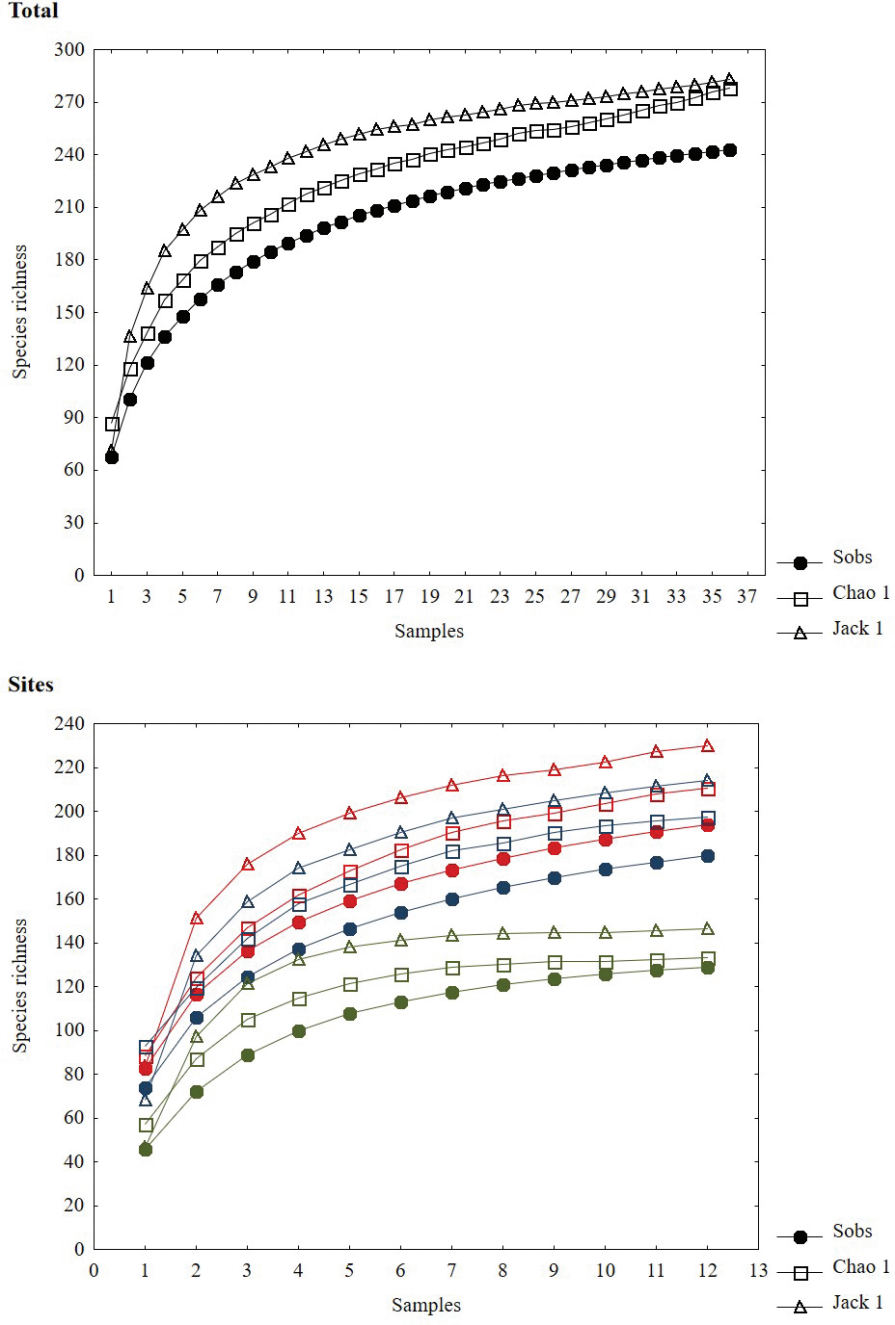
Species accumulation and estimator curves in the Cerro Bufa El Diente, Tamaulipas, Mexico. Upper graphic: accumulation curves for all study area. Lower graphic: Site 1 (red color), Site 2 (blue color) and Site 3 (green color).

**Figure 3.**
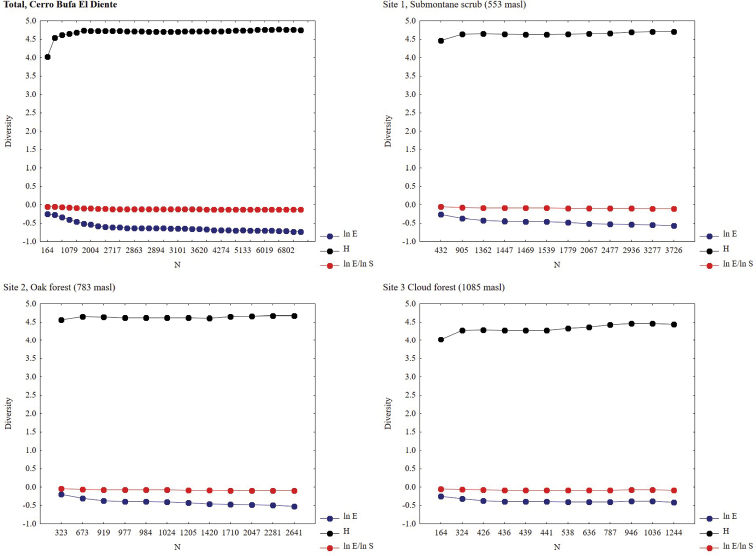
SHE analysis of diversity for the Cerro Bufa El Diente and for each one of altitudinal sites. **H** diversity (Shannon index); ln **E** natural logarithm of evenness; ln **E**/ ln **S** quotient of two previous.

The richness estimators indicated that the total number of butterfly species in the study area was 278 species using Chao 1 and 283 through Jackknife 1(Table 2, Figure 2), suggesting that the observed total of 243 species represents 87.35% (Chao 1) or 85.91% (Jackknife 1) of the actual richness. The data showed a good fit to the linear dependence model (R^2^ = 0.93), with a registered proportion of species of 92.40% and a slope less to 0.1. Total diversity values of Papilionoidea in Cerro Bufa El Diente were 0.98 for the Simpson index and 4.16 for the Shannon index (Table 2). The SHE analysis shows an assemblage with less variation in the natural logarithm of evenness, suggesting a broken stick type distribution (Table 3, Figure 3).

**Table 2.** Richness, abundance and diversity parameters of Papilionoidea in Cerro Bufa El Diente, Tamaulipas, Mexico. Key: S obs = Observed richness; N = Abundance; S est = Estimated richness; LDM = Linear dependence model; R^2^ = LDM determination coefficient; 1-D = Simpson diversity index; H´= Shannon diversity index.

**Ecological parameter**	**Site**			**Season**				**Total**
	**Submontane scrub (553 m asl)**	**Oak forest (783 m asl)**	**Cloud forest (1085 m asl)**	**Dry**		**Rainy**		
				**Early (Dec–Feb)**	**Late (Mar–May)**	**Early (Jun–Aug)**	**Late (Sep–Nov)**	
**S obs** *	194 a	180 a	129 b	65 a	165 b	187 b	207 b	243
N *	3726 a	2641 a	1244 b	297 a	1970 b	2637 b	2707 b	7611
**S est**								
Chao 1	210.67	197.55	133.11	69.33	198.07	208.00	233.46	278.20
Jackknife 1	229.75	213.92	146.42	84.56	204.11	232.33	266.56	282.86
**LDM**								
R^2^	0.96	0.96	0.99	1.00	0.95	0.95	0.94	0.93
S est	219.51	205.61	154.23	95.75	195.55	217.60	243.83	262.99
Slope	0.34	0.39	0.41	1.58	1.04	0.93	1.35	0.04
**Diversity**								
1-D **	0.97 a	0.97 a	0.89 b	0.84 a	0.98 b	0.98 b	0.98 b	0.98
H´ **	4.06 a	3.93 a	3.19 b	2.37 a	4.11 b	4.17 b	4.25 b	4.16

^*^ Values with different letters between columns are significantly different using Kruskal-Wallis and Mann-Whitney Tests: abundance between sites, K= 10.16, DF= 2, *p*= 0.006; richness between sites, K= 7.93, DF= 2, *p*= 0.019; abundance between seasons, K= 21.09, DF= 3, *p*= 0.000, richness between seasons, K= 21.31, DF= 3, *p*= 0.000. ^**^ Diversity values with different letters between columns are significantly different at *p*< 0.05, using permutation tests in R 3.2.3 program.

**Table 3.** SHE analysis to identify the type of abundance distribution of butterflies in Cerro Bufa El Diente, Tamaulipas, Mexico. Marked cells (*) highlight the component with the lowest percentage variation.

**Sites**	**Abundance range**	**ln E**	**H**	**ln E/ln S**	**Distribution**
Site 1, Submontane scrub (553 m asl)	432 to 3726	46.57 *	94.96	51.90	Broken stick
Site 2, Oak forest (783 m asl)	323 to 2641	36.45 *	97.76	39.82	Broken stick
Site 3 Cloud forest (1085 m asl)	164 to 1244	59.83 *	90.65	67.99	Broken stick
Total, Cerro Bufa El Diente	164 to 7611	33.83 *	84.71	43.45	Broken stick

### Altitudinal variation of butterflies

Abundance and number of species of butterflies was significantly different (*p* < 0.05) only between the highest site compared to the intermediate and low sites (Site 3 compared to the Site 2 and 1; Table 2). Both abundance and species richness decreased with increasing altitude (Table 2). In the lowest altitude site, 194 species were recorded which represented between 84.44 andto 92.09% of the estimated richness with the models used. In the second site, the number decreased to 180 species (84.14–91.12% of the estimated) and at the highest site, 129 species were recorded (83.64–96.91% of the estimated) (Figure 2). Determination coefficient in all sites was greater than 0.95, suggesting a good fit of the linear dependence model to the data obtained at each site; contrarily, the slope values was greater than 0.1 in all sites (Table 2).

**Figure 4.**
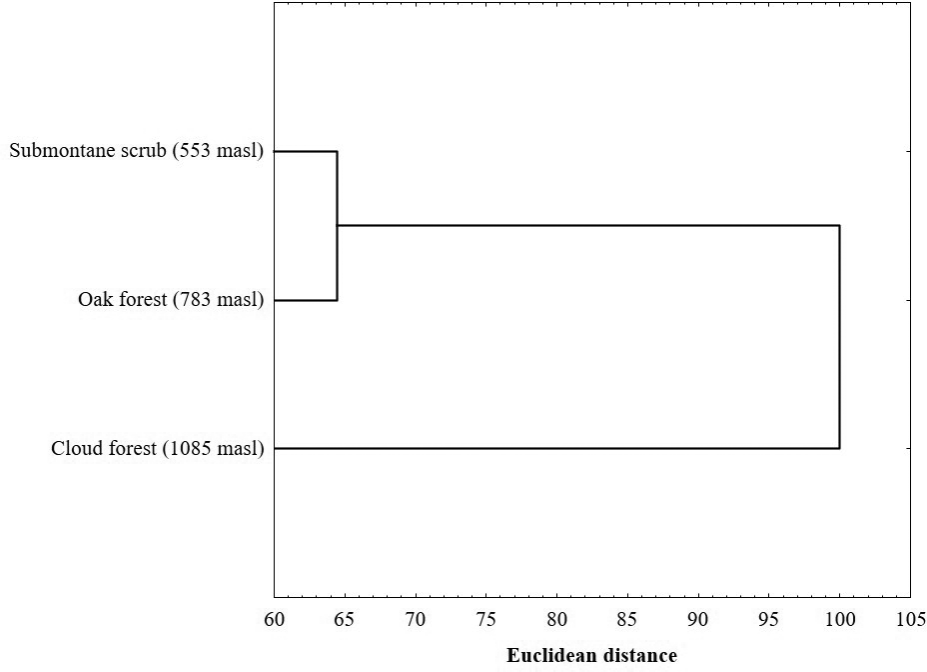
Cluster analysis from sites in the Cerro Bufa El Diente, Tamaulipas, Mexico.

Alpha diversity decreased progressively with increasing altitude and was significantly different between the highest altitude and the other two sites (*p* < 0.05) (Table 2). The result of the SHE analysis for three sites showed a lower variation in natural logarithm of evenness, indicating a broken stick type distribution (Table 3, Figure 3). Of the 243 species recorded in the Cerro Bufa El Diente, 98 were distributed along the entire altitudinal gradient, 64 were recorded only in two sites, and 81 were unique to one of the three sites. Of these 81 unique species, 50 were exclusively from Site 1, 19 for Site 2, and 12 for Site 3 (Appendix 1). The similarity values were greater than 50% between the nearest sites (Site 1 and 2, Site 2 and 3), and less than 50% between the more distant sites (Site 1 and 3). According to the cluster analysis, sites 1 and 2 composed an area of distribution for species that prefer warm climatic conditions, while Site 3 form an independent group of high mountain species (Figure 4).

**Table 4.** Spearman correlations of abundance and richness of butterfly species with climatic factors in Cerro Bufa El Diente, Tamaulipas, Mexico. Marked (*) correlations are significant at *p*< 0.05.

	**Abundance**	**Species richness**
Mean temperature (°C)	0.720 *	0.706 *
Total precipitation (mm)	0.734 *	0.713 *
Solar radiation (kJ)	0.580	0.608 *
Relative humidity (%)	0.748 *	0.664 *

### Seasonal variation of butterflies

**Figure 5.**
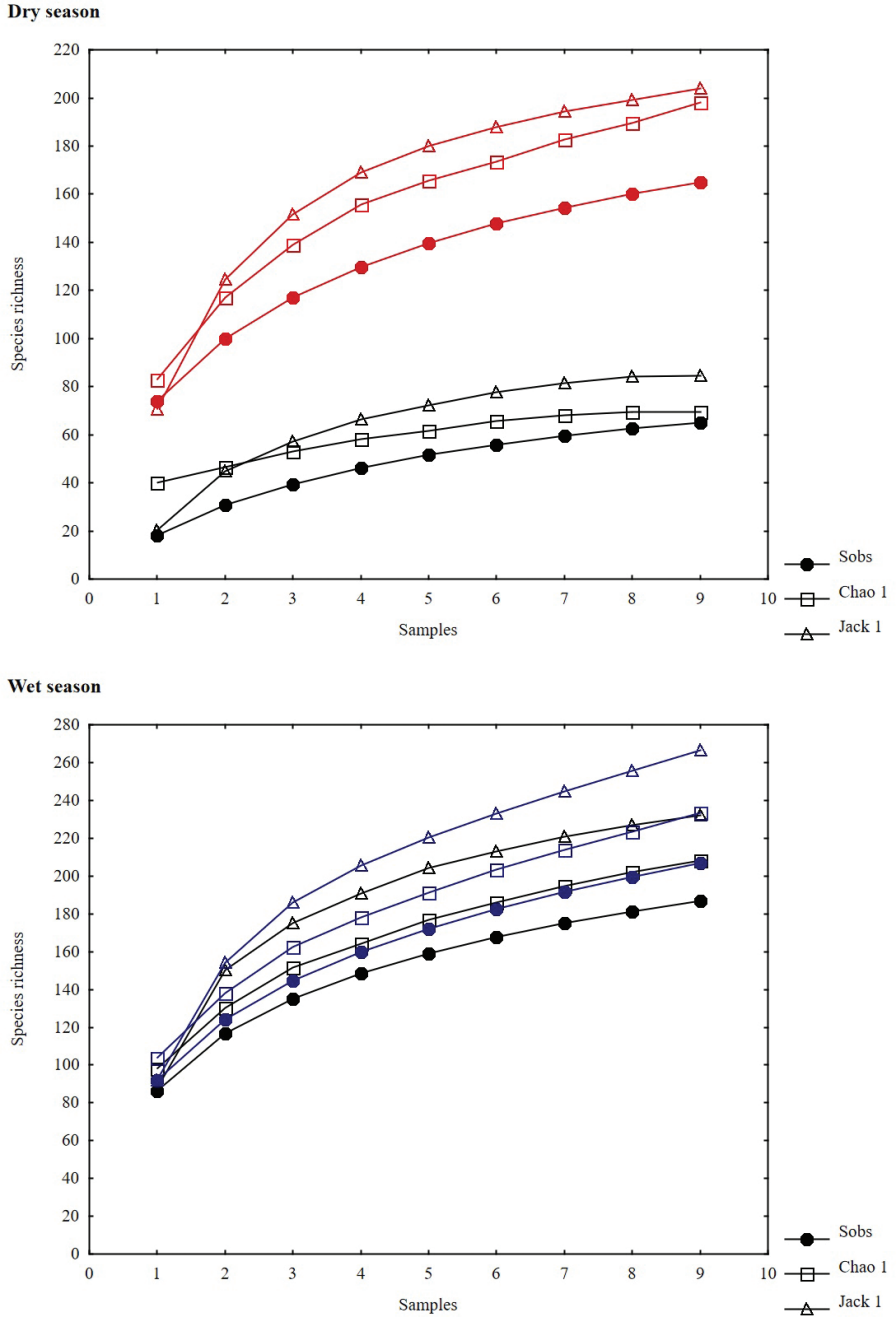
Species accumulation and estimator curves by season in the Cerro Bufa El Diente, Tamaulipas, Mexico. Upper graphic: Early dry season (black color) and late dry season (dark red color). Lower graphic: Early rainy season (black color) and late rainy season (dark blue color).

Differences in abundance and richness of Papilionoidea were found between early dry season and the other three seasons (Table 2, Figure 7). The highest number of specimens was obtained during the rainy season, with 2,707 individuals in the late period and 2,637 in the early one. Lower abundance was found during the late and early dry seasons (1,970 and 297 specimens, respectively) (Table 2). Species richness was higher in the late rainy season, with 207 species representing between 77.66 and 88.67% of estimated richness. Such value decreased to the early dry season (65 species, 67.89 to 93.75%), but increased at the end of dry season (165 species, 80.84 to 84.38%) (Table 2, Figure 5). Determination coefficients of the linear dependence model were higher than 0.90 for all seasons, while the slope values were greater than 0.1 (Table 2).

**Figure 6.**
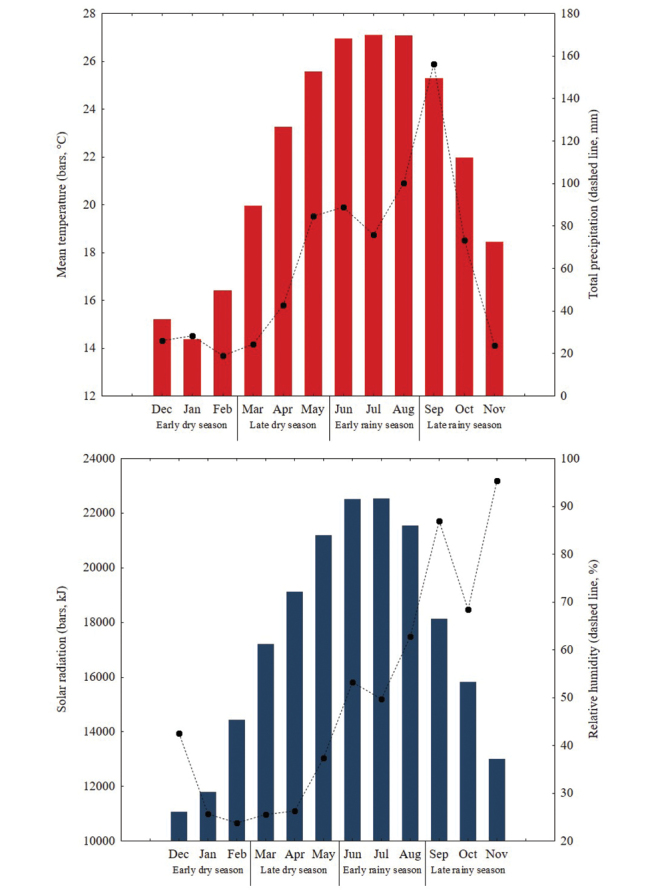
Monthly climate variation in Cerro Bufa El Diente, Tamaulipas, Mexico. Upper graphic: Variation of temperature and precipitation. Lower graphic: Variation of solar radiation and relative humidity.

Highest values of temperature, precipitation, relative humidity and solar radiation were found during both periods of the rainy season (Figure 6). Relative humidity was highly correlated with abundance, while precipitation was better correlated with species richness. Interaction between climate variables compared with the abundance and species richness was positive; however, the correlation between abundance and solar radiation was not significant (Table 4).

According to diversity indices, early dry season was statistically different to the other three seasons (*p* < 0.05) (Table 2). Shannon and Simpson indices indicated the highest diversity during the end of dry season and both periods of the rainy season. Lower diversity was found in early dry season (Table 2). Only 49 species from the total observed, were present during all seasons, 84 were recorded in three seasons, 66 in only two and 44 were exclusive of one season. Of these exclusive species, 19 were recorded at the end of the rainy season, 14 in the early rainy season, 10 at the beginning of the dry season, and only one in the late dry season (Table 2).

According to the Bray-Curtis index, the early and late rainy season had the greatest similarity (80.50%). Rest of the comparisons are above 50%, in the case of the end of the rainy season and the late dry season (72.57%), and from the early rainy season with the late dry season (68.20%), and below 50%, between the beginning and end of the dry season (24.26%), the beginning of the dry and rainy season (19.15%), and the end of the rainy season and the early dry season (19.11%). Cluster analysis shows the formation of two groups, according to the species composition in each season. The first group is composed only of species of the early dry season, and the second group includes species in the late dry season and the beginning and end of the rainy season (Figure 8).

**Figure 7.**
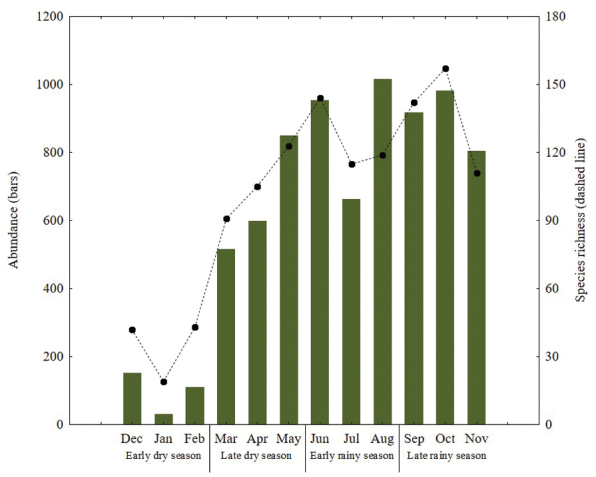
Monthly variation of abundance and richness of butterfly species in Cerro Bufa El Diente, Tamaulipas, Mexico.

**Figure 8.**
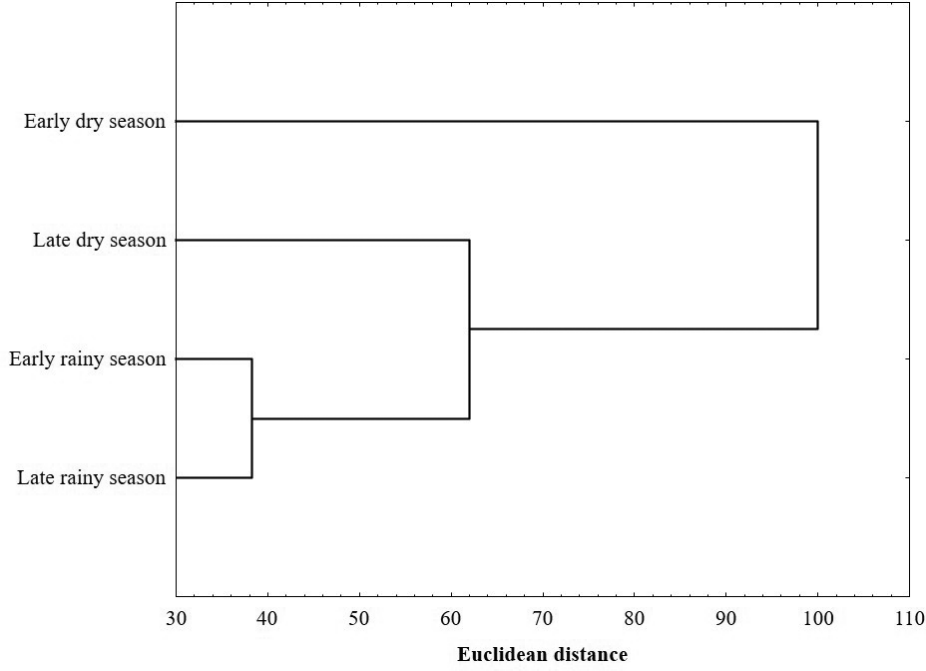
Cluster analysis from seasons in the Cerro Bufa El Diente, Tamaulipas, Mexico.

### Indicator species

The IndVal allowed to quantify the percentage of association for the 243 species in the study area, of which 168 had a higher probability (*p* < 1) of being considered as indicators (Appendix 1). Of these, 66 presented values equal to or greater than 50%, categorizing themselves as detectors or characteristics, while only 19 presented a significant indicator value (*p* < 0.05, Appendix 1). The remaining 75 species had association values equal to or less than 33.33%, with null probabilities (*p* = 1) of being considered as characteristics of a habitat (Appendix 1). The detector species with the highest values of the index were: *Ministrymon azia* (Hewitson, 1873) (70.37%), *Urbanus procne* (Plötz, 1881) (63.89%) and *Chioides zilpa* (Butler, 1872) (57.14%) for the Site 1; *Achlyodes pallida* (R. Felder, 1869) (69.70%), *Smyrna blomfildia datis* Fruhstorfer, 1908 (62.96%) and *Rekoa marius* (Lucas, 1857) (62.32%) for the Site 2; as well as *Amblyscirtes celia* Skinner, 1895 (60.61%) for the Site 3. With respect to the characteristic species, *Microtia elva elva* H. Bates, 1864 (94.23%), *Chlosyne theona bollii* (WH Edwards, 1877) (90.91%) *Heraclides anchisiades idaeus* Fabricius, 1793 (86.67%) presented the highest values for the Site 1; *Oarisma edwardsii* (W. Barnes, 1897) (83.33%) and *Quinta cannae* (Herrich-Schäffer, 1869) (80.00%) were characteristic species of the Site 3 (Appendix 1).

## Discussion

### Faunistic inventory and biodiversity of butterflies in Cerro Bufa El Diente

In Cerro Bufa El Diente, the superfamily Papilionoidea consists of 243 species that represent 69.43% of the richness recorded for Tamaulipas (García 2005; García et al. 2009), and 11.86% in relation Mexico (Warren 2000; Llorente et al. 2006). Hesperiidae are the family with greatest richness, which represents 75.68% of the diversity of the family for the State, and 9.78% in comparison with that of the country. The abundance and species richness by families found in this study is very different when compared to research conducted in other parts of the country. This is due to the specific biotic and abiotic characteristics of each ecoregion, which allow the development of a particular type of fauna (Espinosa and Ocegueda 2008), in this case of the butterflies. This also may be occurring at the species level, where the characteristics of the area, as well as the presence and abundance of its host plants will determine the dominant species (Luis and Llorente 1990; Vargas et al. 1994).

When comparing results found in this research with the few systematic and rigorously sampled inventories of Papilionoidea in Mexico, it can be observed that the species richness in the present study area is high. De la Luz and Madero (2011) in collaboration with the North American Butterfly Association listed 266 species for the state of Nuevo Leon. Luna-Reyes et al. (2010) recorded 145 species for the Lobos Canyon, Yautepec, Morelos state. In the same way, Peña-Morales (2009) listed 120 for two fragments of tropical deciduous forest from the state of Tamaulipas, one in Gómez Farías and the other in Victoria. Luna-Reyes et al. (2008) recorded 142 species for the Huautla mountain range, in the states of Morelos and Puebla. Hernández-Mejía et al. (2008) listed 213 species for Malinalco, State of Mexico. Luna-Reyes and Llorente (2004) listed 85 species for four entities that comprise the Sierra Nevada. Díaz-Batres et al. (2001) recorded 55 species in La Michilía, Durango state. Bizuet-Flores et al. (2001) obtained 69 species for El Chico National Park, Hidalgo state. Luis and Llorente (1993) listed 161 species for Omiltemi Park, Guerrero. Balcázar (1993) presented 205 species for Pedernales, Michoacán. Luis and Llorente (1990) recorded 65 species for the Dinamos, Magdalena Contreras, D.F. Beutelspacher (1982) listed 141 species for El Chorreadero, Chiapas. Considering that these authors used sampling methodologies similar to our study, it can be suggested that Cerro Bufa El Diente is a very important area for distribution and diversity of Rhopalocera in Tamaulipas and Mexico.

Richness estimators suggested that the diurnal butterfly fauna was obtained almost entirely in the Cerro Bufa El Diente, although it is possible that there are still some species to be recorded. In this regard, several authors point out that the increase in number of samples and time of study, or selection of other sampling methods, can aid in complementing faunistic inventories (Sparrow et al. 1994; Daily and Ehrlich 1995; DeVries et al. 1997, 1999; Hughes et al. 1998; Caldas and Robbins 2003; Jiménez et al. 2004; Romo and García 2005; Sackmann 2006; Hernández-Mejía et al. 2008; Bonebrake and Sorto 2009; Pedraza et al. 2010; Álvarez-García et al. 2016; González-Valdivia et al. 2016). However, the critical value in which a faunal inventory can be considered as reliable or complete is from 70% representativeness (observed richness in relation to estimated richness), since above that limit, the number of samples required to register all of the species increases remarkably and disproportionately (Jiménez and Hortal 2003). Taking into account the high percentage of representativeness obtained in this study, it would be necessary to conduct a large number of additional samples only to record a minimum number of possible missing species, since these are considered as accidental species that come from adjacent sites (Thomas 1994; Pozo et al. 2005; Hortal et al. 2006).

Comparing the number of species between different habitats is often enough to give a rapid assessment of a biodiversity measure. However, it is necessary to resort to the use of other statistical measures in order to make comparisons with other studies (Magurran 2004). In this investigation, quantification of diversity was done mainly by the values obtained from Shannon (4.16) and Simpson (0.98). The diversity index of Simpson gives a greater weight to the abundant species and underestimate rare ones, returning values between 0 (low diversity) to a maximum of 1- 1/ S (Moreno 2001). Values of the Shannon index are usually between 1.5 and 3.5, rarely surpassing a value of 4 in very diverse communities (Margalef 1972). This suggests that diversity of butterflies in the study area is actually very high. Moreover, observed values were higher than the diversity present in some tropical communities, where the existing conditions favors a high number of species and individuals, as observed in Montero and Ortiz (2013) for Tablazo Paramo, Cundinamarca, and Camero et al. (2007) in Combeima River, department of Tolima, both in Colombia, and who obtained a total Shannon value of 3.9 for each zone. Accordingly, the broken stick distribution proposed by MacArthur (1960), corroborates ecology heterogeneity of Cerro Bufa El Diente according to the SHE analysis, corresponds biologically to communities of species that colonize and distribute resources at random. In this type of distribution, the most common species are more susceptible to being invaded by the colonizing species than the rare species, resulting in a more equitable community (Gotelli and Graves 1996). The opposite of this distribution is the geometric series, since it reflects the lowest equity among the species of a community (Tokeshi 1990; Fattorini 2006).

On the other hand, the community structure of butterflies may represent evidence of the ecological characteristics of the study area, as a semi-preserved habitat. Community was formed by a moderate group of frequent species and few rare species, which is a characteristic pattern of areas with intermediate ecological quality. On the contrary, and according to Pedraza et al. (2010), a locality with excellent ecological quality is characterized by an assemblage with few frequent species, and a large number of scarce species. All this evidence agrees with previous values of ecological integrity obtained for Sierra de San Carlos (Arriaga et al. 2000). In addition, species that according to Pozo et al. (2005) and Raguso and Llorente (1991) are considered as indicators of disturbed habitats, were present in the study area.

### Elevational effects on diversity patterns of butterflies

Altitude is a variable frequently related to changes in species richness and abundance (Janzen 1993), producing changes in distribution patterns along altitudinal gradients (Llorente 1984; Andrade-Correa 2002), which was demonstrated in this study. In general, a negative correlation of altitude was observed with abundance or species richness; that is, a reduction in the number of specimens and species as the altitudinal gradient increases. According to Andrade-Correa (2002), it is observed that diversity and percentage of exclusive species decrease towards higher altitude areas. Moreover, Hernández-Mejía et al. (2008) states that the overall tendency of richness and abundance is to decrease with the altitudinal gradient. Although each family shows a different rate of decline, Nymphalidae decreases faster, which may be because of their higher number of species accentuates the altitudinal effect. Contrarily the Pieridae family comprises many eurioic species, and therefore the change in richness is almost imperceptible as the altitude increases. In relation to the general abundance of each family, it can be observed how this decreased notably with the increase in altitude. This pattern in the number of individuals has been observed in other studies with butterflies (Luis and Llorente 1990; Vargas et al. 1994, 1999; Andrade-Correa 2002; Luna-Reyes and Llorente 2004; Palacios and Constantino 2006; Camero et al. 2007; Hernández et al. 2008; Ospina et al. 2010; De León 2012; Carrero et al. 2013), as well as in different groups of insects, such as the necrophilous entomofauna (Sánchez et al. 1993) and Scarabaeoidea beetles (Morón 1994).

The variation found in the community patterns could be originate in the abiotic factors that are modified along the altitudinal gradient, such as the air pressure (which decreases with the increase in altitude), solar radiation and precipitation (both increase with the increase in elevation) (McCain and Grytnes 2010), as well as the increase of unfavorable environments and the reduction in availability of resources (Camero 2003; Camero et al. 2007). This can favor some species in particular, allowing them to increase their abundance at a certain altitudinal level, as was observed in the case of the species categorized as indicators, three for the last altitudinal site, three at the intermediate site and 13 for the first. In addition, the available area that species can occupy decreases with altitude (McCain and Grytnes 2010), which may cause a reduction in the number of individuals per species in higher sites (Camero 2003; Camero et al. 2007). Besides, the linear decrease in temperature, which decreases on average 0.68 °C per 100 meters of increase in elevation, is maybe one of the most important abiotic factors in the altitudinal distribution of species (McCain and Grytnes 2010). Therefore, the lower abundance in the higher altitude site could be related to its lower temperature, which represent an unfavorable factor for these insects (Kremen et al. 1993; Fagua 1999). The importance of this variable has also been observed in other studies of Lepidoptera (Luis and Llorente 1990; Vargas et al. 1994, 1999; Luna-Reyes and Llorente 2004; Hernández et al. 2008; De León 2012).

Vegetation is another factor of great influence for butterfly composition along altitudinal gradients (Llorente 1984; Luis et al. 2000). In the study area, the first altitudinal site corresponds to submontane scrub, which shows a high density of plants in the herbaceous and shrub layers (Briones 1991; Martínez 1998). Such condition represents a greater availability of food resources, allowing the increase in abundance of diurnal Lepidoptera in this area. On the other hand, the intermediate altitudinal site represents a transitional zone between the Papilionoidea fauna of the submontane scrub and the fauna of the cloud forest, which was corroborated with the Bray-Curtis index. In relation to this, the lower similarity between the extreme sites of the altitudinal gradient would be determined by the distance between both zones. In addition, the fact that the percentages of similarity were mostly greater than 50%, establishes that the compositions of the communities in the study area are similar in each site, this maybe because the Sierra de San Carlos and especially the rocky massif Bufa El Diente represent ideal sites for research on biodiversity over a period of a year due to its small area (Martínez 1998).

According to the behavior of both variables, abundance and diversity in the different sites, it can be suggested that vegetation and perhaps temperature and humidity are the determining factors in the abundance and richness of species of butterflies in the study area, parameters that decrease with altitude. Protecting populations of Papilionoidea in mountain areas, often depends on the conservation of lower adjacent areas, where the greatest abundance may occur (Andrade-Correa 2002). Another issue directly associated with the conservation of the populations, is that middle and high mountain areas are frequently used as natural corridors in the migration of butterfly species (Monteagudo et al. 2001). It is also necessary to take into account the displacements that occur from the lower parts towards the high elevation areas, because species search for foraging sites and better climatic conditions (Bonebrake et al. 2010). Therefore, biodiversity inventories along an altitudinal gradient, such as the one carried out in this research, serve as monitoring studies of habitat quality, which allows identifying important areas in conservation and management policies (Dewenter and Teja 2000; Hoyle and Harbone 2005; Fattorini 2006).

### Seasonal effects on diversity patterns of butterflies

In general, the pattern of monthly variation of abundance and species richness was similar to the results reported by Luis and Llorente (1990), Luis et al. (1991), Vargas et al. (1994), Hernández-Mejía et al. (2008), Luna et al. (2008), Pozo et al. (2008) and Luna et al. (2010). From March to November, the greatest number of species and specimens was recorded, with lower values between December and February, the first months of the dry season. As for the Shannon index, both the end of the dry season as early and late wet season had the highest values, which were above 4.0, and so they represent a high diversity (Margalef 1972). In addition, the Bray-Curtis index and Cluster analysis indicated that late dry season and two periods of the wet season had a very different faunal composition compared to the early dry season. Therefore, the most favorable flight period for butterflies in the study area occurs during the last months of the dry season and the months corresponding to the wet season.

Seasonality is a very important factor in species distribution, being of great relevance for insects, since they cannot regulate their body temperature and therefore require favorable environmental conditions for metabolic activities and development of their life cycles (Brown 1984; Morón and Terrón 1984; Wolda 1988). Among the microclimatic factors that influence the seasonal distribution of butterflies in Cerro Bufa El Diente are the temperature and relative humidity. This temporal association is commonly recorded in tropical areas (Arteaga 1991; Luis et al. 1991; Vargas et al. 1992, 1999; Balcázar 1993), in which the imagos are most active during the early and late wet seasons, that is, when the availability of resources is greater, wintering in diapause (Scott 1979; Courtney 1986).

Additionally, butterflies are closely associated to plants, and their presence depends on the flora and structure of the vegetation (Shapiro 1974). Thus, it is possible that the wetter conditions in June to November favored the increase of diversity and biomass of the plant community, which can lead to the establishment of more species and larger populations of butterflies (Rhoades 1983). Temperature is more stable in this period, but humidity conditions are contrasting and remarkably superior with respect to the dry season, in which the total precipitation is 225 mm, while that in the rainy season is 518.7 mm. Although the first rains take place towards the end of May, the greater precipitation occurs from September to November, and as a consequence there is greater cloudiness that reduces evaporation. During this season, vegetation diversity and density increases, thus providing a greater amount of resources that are used by butterflies for their feeding, oviposition and protection, which favor the presence of more species with larger populations. Besides, the presence of rainfall correlates directly with abundance and richness of insects (Wolda 1988), since it affects the physiology of the reproduction, the ontogenetic development and the behavior of the imagoes; indirectly, it can also affect populations because of its effects on plant phenology (Vargas et al. 1999). As in other studies, the late wet season would represent the period where the greatest number of Lepidoptera species complete their diapause stage and begin their feeding, reproduction and oviposition stage (Owen 1971; Wolda 1988).

On the contrary, the highest variation in temperature as well as the highest number of clear days occur during the months of November to April, leading to high evaporation rates. Under these conditions, most of the vegetation is dry, especially some herbaceous plants that, when flowering, provide food for imagoes. During the drought period, water reserves of tree and shrub species are also reduced, modifying their growth, nectar production, nutritional content, or even texture and turgor of leaves, which constitute food resources for most lepidoptera species. Therefore, although trees and shrubs are present in the habitat, many of them cannot be used by butterflies during this period due to their deciduous phenology, affecting in this way the community composition and populations of butterflies in these months. In addition, some compounds present in plants can vary in each season and not be palatable in certain months, so they are not nutritious for the immature stages of many species. Nevertheless, it is possible that the species are in diapause during the cold months (Scott 1979)

The results obtained in this work may have implications for the conservation of biodiversity, mainly butterflies, as they provide information to build a research line focused on detecting the effects of climatic variations on the composition of species and providing an approximation of the behavior of its diversity. In the particular case of diurnal Lepidoptera, the impact of climate change on populations can be measured by monitoring the temporary replacement of the composition of species in the community and the environmental gradients of temperature and relative humidity. This information can be used in the evaluation and use of environmental services by pollination of a large variety of plants, which is carried out by Lepidoptera (Grøtan et al. 2012, 2014; Checa et al. 2014; Forrest 2016).

## Conclusions

For the first time in northeastern Mexico, the Papilionoidea group was systematically sampled during an annual sampling period. A total of 7,611 specimens belonging to six families, 20 subfamilies, 32 tribes, 148 genera, and 243 species of butterfly was collected from the study area. The highest abundance and richness of species, as well as alpha diversity was recorded in the lowest elevation site, and decreases significantly with increasing altitude, the tendency of altitudinal distribution of the Papilionoidea butterflies in Cerro Bufa El Diente is well defined to the environmental characteristics of the lower zone, agreeing with the Rapoport rule. The sites of low and intermediate altitude constitute an area of distribution of tropical species, while the site of the third floor forms an independent group of high mountain species, according to the conglomerate analysis carried out.

The greatest abundance and richness of species, as well as alpha diversity, was obtained during the late wet season, decreasing towards the beginning of the dry season. The geographical location of the study area plus the different plant compositions of the three sampled sites could be the main reason for the variation found here in the butterfly communities with altitude and season. In addition, relative humidity and temperature can influence the community of Rhopalocera in the study area; however, both abiotic factors directly affect plant composition, which is assumed to be the main factor in determining the composition and abundance of butterfly species.

This work is one of the first studies of diurnal butterflies in a specific area of northeastern Mexico, in which altitude and season are analyzed. The information presented here provides reference data that allow the comparison of the diversity and richness of Papilionoidea species at a regional and national scale. This information could be used as an initial step to analyze the possible use of butterflies as a biodiversity indicator group in Mexico.

## Appendix 1

**Table:** Taxonomic list of Papilionoidea by season and site in Cerro Bufa El Diente, Tamaulipas, Mexico. Abundance (upper row) and indicator values (lower row) are indicated for each species. N = Total abundance; 1 = Submontane scrub, 553 m asl; 2 = Oak forest, 783 m asl; 3 = Cloud forest, 1085 m asl; marked species (*) had a significant indicator value at *p* < 0.05.

Taxon	Dry Season						Rainy Season						N
	Early (Dec-Feb)			Late (Mar-May)			Early (Jun-Aug)			Late (Sep-Nov)			
	1	2	3	1	2	3	1	2	3	1	2	3	
**Papilionidae Latreille, 1802**													
**Papilioninae Latreille, 1802**													
**Troidini Talbot, 1939**													
*Battus philenor philenor* (Linnaeus, 1771)	0	0	0	9	11	6	2	2	3	5	2	3	43
	0.0	0.0	0.0	20.9	25.6	14.0	1.6	1.6	2.3	3.9	1.6	2.3	73.6
*Battus polydamas polydamas* (Linnaeus, 1758)	1	1	0	2	4	3	9	10	6	7	6	6	55
	0.6	0.6	0.0	1.2	2.4	1.8	16.4	18.2	10.9	12.7	10.9	10.9	86.7
**Leptocircini W. F. Kirby, 1896**													
*Protographium epidaus epidaus* (Doubleday, 1846)	0	0	0	1	0	0	0	0	0	2	0	0	3
	0.0	0.0	0.0	11.1	0.0	0.0	0.0	0.0	0.0	22.2	0.0	0.0	33.3
*Protographium philolaus philolaus* (Boisduval, 1836)	0	0	0	3	1	2	5	2	3	2	1	1	20
	0.0	0.0	0.0	5.0	1.7	3.3	16.7	6.7	10.0	3.3	1.7	1.7	50.0
**Papilionini Latreille, 1802**													
*Papilio polyxenes asterius* (Stoll, 1782)	2	1	0	8	5	3	6	5	5	8	9	5	57
	1.2	0.6	0.0	14.0	8.8	3.5	10.5	8.8	5.8	14.0	15.8	8.8	91.8
*Pterourus alexiares garcia* Rothschild & Jordan, 1906	0	0	0	0	0	6	0	0	0	0	1	4	11
	0.0	0.0	0.0	0.0	0.0	36.4	0.0	0.0	0.0	0.0	3.0	12.1	51.5
*Pterourus pilumnus* Boisduval, 1836	0	0	0	4	4	2	4	6	4	6	3	3	36
	0.0	0.0	0.0	7.4	7.4	3.7	7.4	16.7	11.1	11.1	5.6	5.6	75.9
*Pterourus palamedes leontis* Rothschild & Jordan, 1906	0	0	0	0	0	0	0	3	0	2	1	0	6
	0.0	0.0	0.0	0.0	0.0	0.0	0.0	33.3	0.0	11.1	5.6	0.0	50.0
*Pterourus garamas abderus* Höpffer, 1856	0	0	0	0	1	4	0	0	6	0	0	7	18
	0.0	0.0	0.0	0.0	1.9	14.8	0.0	0.0	33.3	0.0	0.0	38.9	88.9
*Pterourus victorinus victorinus* E. Doubleday, 1844	0	0	0	0	0	3	0	0	0	0	0	2	5
	0.0	0.0	0.0	0.0	0.0	40.0	0.0	0.0	0.0	0.0	0.0	13.3	53.3
*Heraclides cresphontes* Cramer, 1777	1	1	0	0	0	0	19	14	11	25	12	6	89
	0.4	0.4	0.0	0.0	0.0	0.0	21.3	15.7	12.4	28.1	13.5	6.7	98.5
*Heraclides astyalus pallas* G. Gray, 1853	0	0	0	1	0	0	3	1	0	1	0	0	6
	0.0	0.0	0.0	5.6	0.0	0.0	16.7	5.6	0.0	5.6	0.0	0.0	33.3
*Heraclides ornythion* Boisduval, 1836	0	0	0	0	0	0	13	5	0	5	3	0	26
	0.0	0.0	0.0	0.0	0.0	0.0	33.3	12.8	0.0	12.8	7.7	0.0	66.7
**Heraclides anchisiades idaeus* Fabricius, 1793	0	0	0	8	1	0	0	0	0	5	1	0	15
	0.0	0.0	0.0	53.3	2.2	0.0	0.0	0.0	0.0	33.3	2.2	0.0	91.1
**Pieridae Swainson, 1820**													
**Coliadinae Swainson, 1821**													
*Kricogonia lyside* (Godart, 1819)	4	2	0	14	8	5	28	24	20	19	21	14	159
	1.7	0.8	0.0	8.8	5.0	3.1	17.6	15.1	12.6	11.9	13.2	8.8	98.7
*Nathalis iole iole* Boisduval, 1836	2	1	0	5	3	8	1	3	3	6	6	10	48
	1.4	0.7	0.0	10.4	6.3	16.7	0.7	2.1	2.1	12.5	12.5	20.8	86.1
*Eurema daira eugenia* (Wallengren, 1860)	0	0	0	0	0	0	2	0	0	1	0	0	3
	0.0	0.0	0.0	0.0	0.0	0.0	22.2	0.0	0.0	11.1	0.0	0.0	33.3
*Eurema boisduvaliana* (C. Felder & R. Felder, 1865)	0	0	0	0	0	0	1	0	0	3	0	0	4
	0.0	0.0	0.0	0.0	0.0	0.0	8.3	0.0	0.0	50.0	0.0	0.0	58.3
*Eurema mexicana mexicana* (Boisduval, 1836)	1	1	0	8	6	2	4	5	2	10	5	2	46
	0.7	0.7	0.0	17.4	13.0	2.9	5.8	7.2	1.4	21.7	10.9	1.4	83.3
*Eurema salome jamapa* (Reakirt, 1866)	0	0	0	0	0	0	1	2	0	2	0	0	5
	0.0	0.0	0.0	0.0	0.0	0.0	6.7	13.3	0.0	13.3	0.0	0.0	33.3
*Abaeis nicippe* (Cramer, 1779)	0	0	0	6	8	3	13	5	7	4	5	1	52
	0.0	0.0	0.0	7.7	10.3	1.9	25.0	9.6	13.5	5.1	6.4	0.6	80.1
*Pyrisitia proterpia* (Fabricius, 1775)	4	4	0	6	7	3	9	6	4	8	3	0	54
	7.4	4.9	0.0	11.1	13.0	3.7	16.7	11.1	4.9	14.8	3.7	0.0	91.4
*Pyrisitia lisa centralis* (Herrich-Schäffer, 1865)	5	4	0	12	10	3	12	8	9	12	10	6	91
	5.5	2.9	0.0	13.2	11.0	2.2	13.2	8.8	9.9	13.2	11.0	4.4	95.2
*Pyrisitia nise nelphe* (R. Felder, 1869)	5	1	0	11	10	7	9	9	2	10	9	4	77
	6.5	0.4	0.0	14.3	13.0	9.1	11.7	7.8	0.9	13.0	11.7	5.2	93.5
*Pyrisitia dina westwoodii* (Boisduval, 1836)	0	0	0	0	0	0	0	0	0	6	3	0	9
	0.0	0.0	0.0	0.0	0.0	0.0	0.0	0.0	0.0	44.4	22.2	0.0	66.7
*Colias eurytheme* Boisduval, 1832	0	0	0	0	0	0	0	0	0	1	0	0	1
	0.0	0.0	0.0	0.0	0.0	0.0	0.0	0.0	0.0	33.3	0.0	0.0	33.3
*Zerene cesonia cesonia* (Stoll, 1790)	1	2	0	2	3	5	20	10	11	31	15	7	107
	0.3	0.6	0.0	0.6	0.9	1.6	18.7	9.3	10.3	29.0	14.0	6.5	91.9
*Anteos clorinde* (Godart, 1824)	0	0	0	0	0	0	1	1	2	1	5	5	15
	0.0	0.0	0.0	0.0	0.0	0.0	2.2	2.2	4.4	2.2	33.3	22.2	66.7
**Anteos maerula* (Fabricius, 1775)	2	0	0	0	0	0	10	6	3	8	5	4	38
	3.5	0.0	0.0	0.0	0.0	0.0	26.3	15.8	5.3	21.1	13.2	7.0	92.1
*Phoebis sennae marcellina* (Cramer, 1777)	4	3	1	19	16	9	15	6	10	19	14	7	123
	2.2	2.4	0.3	15.4	13.0	7.3	12.2	4.9	8.1	15.4	11.4	5.7	98.4
*Phoebis philea philea* (Linnaeus, 1763)	1	0	0	0	0	0	5	7	7	11	4	3	38
	0.9	0.0	0.0	0.0	0.0	0.0	13.2	18.4	12.3	28.9	10.5	5.3	89.5
*Phoebis argante argante* (Fabricius, 1775)	0	0	0	1	2	0	2	3	1	4	3	1	17
	0.0	0.0	0.0	2.0	3.9	0.0	3.9	5.9	2.0	15.7	5.9	2.0	41.2
*Phoebis agarithe agarithe* (Boisduval, 1836)	2	1	3	25	15	12	24	23	14	22	17	9	167
	0.4	0.2	0.6	15.0	9.0	7.2	14.4	13.8	8.4	13.2	10.2	5.4	97.6
**Pierinae Swainson, 1820**													
**Pierini Swainson, 1820**													
*Glutophrissa drusilla tenuis* (Lamas, 1981)	0	0	0	6	7	5	9	5	5	7	5	7	56
	0.0	0.0	0.0	7.1	8.3	6.0	10.7	6.0	6.0	8.3	6.0	8.3	66.7
*Pieriballia viardi viardi* (Boisduval, 1836)	0	0	0	0	0	0	0	0	0	1	0	0	1
	0.0	0.0	0.0	0.0	0.0	0.0	0.0	0.0	0.0	33.3	0.0	0.0	33.3
*Pontia protodice* (Boisduval & Le Conte, 1830)	0	0	0	0	0	0	15	16	20	3	3	4	61
	0.0	0.0	0.0	0.0	0.0	0.0	24.6	26.2	32.8	1.6	1.6	2.2	89.1
*Ascia monuste monuste* (Linnaeus, 1764)	0	0	0	0	0	0	6	5	2	5	7	3	28
	0.0	0.0	0.0	0.0	0.0	0.0	14.3	11.9	2.4	11.9	16.7	3.6	60.7
*Ganyra josephina josepha* (Salvin & Godman, 1868)	0	0	0	3	1	1	8	6	7	12	8	3	49
	0.0	0.0	0.0	2.0	0.7	0.7	16.3	8.2	9.5	24.5	16.3	2.0	80.3
**Lycaenidae Leach, 1815**													
**Theclinae Swainson, 1831**													
**Eumaeini E. Doubleday, 1847**													
*Eumaeus childrenae* (G. Gray, 1832)	0	3	0	3	3	2	17	13	18	8	13	7	87
	0.0	1.1	0.0	1.1	1.1	0.8	19.5	14.9	20.7	9.2	14.9	5.4	88.9
*Atlides halesus corcorani* Clench, 1942	0	0	0	1	0	0	0	0	0	0	0	0	1
	0.0	0.0	0.0	33.3	0.0	0.0	0.0	0.0	0.0	0.0	0.0	0.0	33.3
*Rekoa palegon* (Cramer, 1780)	0	0	0	0	1	0	0	1	0	1	0	0	3
	0.0	0.0	0.0	0.0	11.1	0.0	0.0	11.1	0.0	11.1	0.0	0.0	33.3
**Rekoa marius* (Lucas, 1857)	1	2	0	0	0	0	3	6	0	3	7	1	23
	1.4	5.8	0.0	0.0	0.0	0.0	8.7	26.1	0.0	13.0	30.4	1.4	87.0
*Arawacus jada* (Hewitson, 1867)	0	0	0	0	1	0	0	1	0	1	0	0	3
	0.0	0.0	0.0	0.0	11.1	0.0	0.0	11.1	0.0	11.1	0.0	0.0	33.3
*Ocaria ocrisia* (Hewitson, 1868)	0	0	0	0	0	0	0	0	0	0	1	0	1
	0.0	0.0	0.0	0.0	0.0	0.0	0.0	0.0	0.0	0.0	33.3	0.0	33.3
**Chlorostrymon simaethis sarita* (Skinner, 1895)	0	0	0	0	0	0	3	0	0	14	1	0	18
	0.0	0.0	0.0	0.0	0.0	0.0	5.6	0.0	0.0	77.8	1.9	0.0	85.2
*Cyanophrys herodotus* (Fabricius, 1793)	0	0	0	3	1	1	2	0	0	3	1	0	11
	0.0	0.0	0.0	9.1	3.0	3.0	6.1	0.0	0.0	9.1	3.0	0.0	33.3
*Cyanophrys miserabilis* (Clench, 1946)	0	0	0	1	0	0	3	0	0	1	0	0	5
	0.0	0.0	0.0	6.7	0.0	0.0	40.0	0.0	0.0	6.7	0.0	0.0	53.3
*Cyanophrys longula* (Hewitson, 1868)	0	0	0	0	1	1	0	0	1	0	0	0	3
	0.0	0.0	0.0	0.0	11.1	11.1	0.0	0.0	11.1	0.0	0.0	0.0	33.3
*Callophrys xami texami* Clench, 1981	0	0	0	0	0	1	0	0	0	0	0	0	1
	0.0	0.0	0.0	0.0	0.0	33.3	0.0	0.0	0.0	0.0	0.0	0.0	33.3
*Allosmaitia strophius* (Godart, 1824)	0	0	0	0	0	0	0	0	0	1	0	0	1
	0.0	0.0	0.0	0.0	0.0	0.0	0.0	0.0	0.0	33.3	0.0	0.0	33.3
*Electrostrymon hugon* (Godart, 1824)	0	0	0	1	2	0	3	3	0	2	1	0	12
	0.0	0.0	0.0	2.8	11.1	0.0	8.3	16.7	0.0	5.6	2.8	0.0	47.2
*Electrostrymon guzanta* (Schaus, 1902)	0	0	0	0	0	0	2	0	0	2	1	0	5
	0.0	0.0	0.0	0.0	0.0	0.0	13.3	0.0	0.0	26.7	6.7	0.0	46.7
*Calycopis isobeon* (Butler & H. Druce, 1872)	1	2	0	7	11	1	3	11	3	5	13	1	58
	0.6	2.3	0.0	12.1	19.0	0.6	3.4	12.6	1.7	5.7	14.9	0.6	73.6
*Strymon melinus melinus* Hübner, 1818	2	0	0	5	5	3	22	15	5	8	5	1	71
	0.9	0.0	0.0	2.3	2.3	1.4	31.0	21.1	7.0	7.5	4.7	0.5	78.9
*Strymon rufofusca* (Hewitson, 1877)	2	0	0	0	0	0	1	0	0	4	0	0	7
	9.5	0.0	0.0	0.0	0.0	0.0	4.8	0.0	0.0	38.1	0.0	0.0	52.4
*Strymon albata* (C. Felder & R. Felder, 1865)	0	0	0	0	0	0	1	0	0	0	0	0	1
	0.0	0.0	0.0	0.0	0.0	0.0	33.3	0.0	0.0	0.0	0.0	0.0	33.3
*Strymon alea* (Godman & Salvin, 1887)	0	0	0	1	0	0	4	0	0	8	0	0	13
	0.0	0.0	0.0	2.6	0.0	0.0	20.5	0.0	0.0	41.0	0.0	0.0	64.1
*Strymon bebrycia* (Hewitson, 1868)	0	0	0	1	0	0	0	0	0	2	0	0	3
	0.0	0.0	0.0	11.1	0.0	0.0	0.0	0.0	0.0	44.4	0.0	0.0	55.6
*Strymon yojoa* (Reakirt, 1867)	1	0	0	0	0	0	0	0	0	0	0	0	1
	33.3	0.0	0.0	0.0	0.0	0.0	0.0	0.0	0.0	0.0	0.0	0.0	33.3
*Strymon bazochii bazochii* (Godart, 1824)	0	0	0	0	0	0	4	2	0	5	1	0	12
	0.0	0.0	0.0	0.0	0.0	0.0	22.2	11.1	0.0	41.7	2.8	0.0	77.8
*Strymon istapa istapa* (Reakirt, 1867)	1	1	0	10	6	3	6	10	1	14	7	1	60
	0.6	0.6	0.0	16.7	10.0	1.7	10.0	16.7	0.6	23.3	11.7	0.6	92.2
*Strymon serapio* (Godman & Salvin, 1887)	0	0	0	0	0	0	0	1	0	0	0	0	1
	0.0	0.0	0.0	0.0	0.0	0.0	0.0	33.3	0.0	0.0	0.0	0.0	33.3
*Ministrymon clytie* (W. H. Edwards, 1877)	3	0	0	4	5	1	9	10	3	9	7	1	52
	3.8	0.0	0.0	5.1	6.4	0.6	17.3	19.2	3.8	17.3	13.5	0.6	87.8
**Ministrymon azia* (Hewitson, 1873)	0	0	0	5	0	0	2	0	0	2	0	0	9
	0.0	0.0	0.0	55.6	0.0	0.0	7.4	0.0	0.0	7.4	0.0	0.0	70.4
*Strephonota tephraeus* (Geyer, 1837)	0	0	0	0	0	0	0	0	0	0	1	0	1
	0.0	0.0	0.0	0.0	0.0	0.0	0.0	0.0	0.0	0.0	33.3	0.0	33.3
*Panthiades bathildis* (C. Felder & R. Felder, 1865)	0	0	0	0	0	0	0	0	0	1	0	0	1
	0.0	0.0	0.0	0.0	0.0	0.0	0.0	0.0	0.0	33.3	0.0	0.0	33.3
*Parrhasius moctezuma* (Clench, 1971)	0	0	0	0	0	0	0	1	0	1	1	0	3
	0.0	0.0	0.0	0.0	0.0	0.0	0.0	11.1	0.0	11.1	11.1	0.0	33.3
**Polyommatinae Swainson, 1827**													
*Leptotes cassius cassidula* (Boisduval, 1870)	6	2	0	18	12	6	15	7	2	13	12	5	98
	6.1	1.4	0.0	18.4	12.2	6.1	15.3	7.1	0.7	13.3	12.2	3.4	96.3
*Leptotes marina* (Reakirt, 1868)	0	0	0	9	3	0	11	5	0	11	5	0	44
	0.0	0.0	0.0	13.6	4.5	0.0	25.0	7.6	0.0	16.7	7.6	0.0	75.0
*Brephidium exilis exilis* (Boisduval, 1852)	0	0	0	0	0	0	1	0	0	0	0	0	1
	0.0	0.0	0.0	0.0	0.0	0.0	33.3	0.0	0.0	0.0	0.0	0.0	33.3
*Zizula cyna* (W. H. Edwards, 1881)	0	0	0	2	0	0	2	0	0	1	0	0	5
	0.0	0.0	0.0	26.7	0.0	0.0	13.3	0.0	0.0	6.7	0.0	0.0	46.7
*Cupido comyntas comyntas* (Godart, 1824)	0	0	0	1	0	0	0	0	0	0	0	0	1
	0.0	0.0	0.0	33.3	0.0	0.0	0.0	0.0	0.0	0.0	0.0	0.0	33.3
*Celastrina ladon* (Cramer, 1780)	0	0	1	1	0	1	0	0	0	0	0	2	5
	0.0	0.0	6.7	6.7	0.0	6.7	0.0	0.0	0.0	0.0	0.0	13.3	33.3
*Echinargus isola* (Reakirt, 1867)	0	0	0	15	10	7	16	9	4	16	10	5	92
	0.0	0.0	0.0	16.3	10.9	7.6	17.4	9.8	2.9	17.4	10.9	3.6	96.7
**Hemiargus ceraunus astenidas* (Lucas, 1857)	2	0	0	15	7	1	9	4	1	10	9	3	61
	2.2	0.0	0.0	24.6	11.5	0.5	9.8	4.4	0.5	16.4	14.8	3.3	88.0
**Riodinidae Grote, 1895**													
**Euselasiinae Kirby, 1871**													
*Euselasia eubule* (R. Felder, 1869)	0	0	0	0	0	0	0	1	0	0	0	0	1
	0.0	0.0	0.0	0.0	0.0	0.0	0.0	33.3	0.0	0.0	0.0	0.0	33.3
**Riodininae Grote, 1895**													
**Calephelis nemesis australis* (W. H. Edwards, 1877)	0	0	0	3	1	1	16	9	3	11	9	2	55
	0.0	0.0	0.0	1.8	0.6	0.6	29.1	16.4	3.6	20.0	16.4	1.2	89.7
*Calephelis perditalis perditalis* W. Barnes & McDunnough, 1918	0	0	0	16	12	5	14	7	2	15	12	3	86
	0.0	0.0	0.0	18.6	14.0	5.8	16.3	8.1	0.8	17.4	14.0	2.3	97.3
*Calephelis rawsoni* McAlpine, 1939	0	0	0	0	0	0	1	0	0	0	0	0	1
	0.0	0.0	0.0	0.0	0.0	0.0	33.3	0.0	0.0	0.0	0.0	0.0	33.3
*Caria ino melicerta* Schaus, 1890	0	0	0	10	6	1	10	6	1	9	5	1	49
	0.0	0.0	0.0	20.4	12.2	0.7	20.4	8.2	0.7	18.4	10.2	0.7	91.8
*Lasaia sula peninsularis* Clench, 1972	0	0	0	0	0	0	0	0	0	3	0	0	3
	0.0	0.0	0.0	0.0	0.0	0.0	0.0	0.0	0.0	33.3	0.0	0.0	33.3
*Emesis tenedia* C. Felder & R. Felder, 1861	2	0	0	0	0	0	3	7	4	4	9	7	36
	1.9	0.0	0.0	0.0	0.0	0.0	2.8	13.0	11.1	7.4	25.0	19.4	80.6
*Emesis emesia* (Hewitson, 1867)	2	1	0	0	0	0	9	5	1	14	12	6	50
	1.3	0.7	0.0	0.0	0.0	0.0	18.0	6.7	0.7	28.0	24.0	12.0	91.3
*Apodemia hypoglauca hypoglauca* (Godman & Salvin, 1878)	0	0	0	0	0	1	0	0	1	0	1	0	3
	0.0	0.0	0.0	0.0	0.0	11.1	0.0	0.0	11.1	0.0	11.1	0.0	33.3
*Apodemia walkeri* Godman & Salvin, 1886	0	0	0	0	0	0	0	0	0	0	1	0	1
	0.0	0.0	0.0	0.0	0.0	0.0	0.0	0.0	0.0	0.0	33.3	0.0	33.3
**Nymphalidae Rafinesque, 1815**													
**Libytheinae Boisduval, 1833**													
*Libytheana carinenta larvata* (Strecker, 1878)	0	0	0	27	13	5	56	33	18	37	16	8	213
	0.0	0.0	0.0	8.5	4.1	0.8	26.3	15.5	8.5	17.4	7.5	2.5	90.9
**Danainae Boisduval, 1833**													
**Danaini Boisduval, 1833**													
*Danaus plexippus plexippus* (Linnaeus, 1758)	0	0	0	2	3	3	0	0	0	3	3	9	23
	0.0	0.0	0.0	2.9	4.3	4.3	0.0	0.0	0.0	8.7	8.7	26.1	55.1
*Danaus gilippus thersippus* (H. Bates, 1863)	5	2	0	12	7	3	14	10	5	12	10	6	86
	5.8	1.6	0.0	14.0	8.1	2.3	16.3	11.6	3.9	14.0	11.6	7.0	96.1
*Danaus eresimus montezuma* Talbot, 1943	2	0	0	0	0	0	5	2	1	14	9	6	39
	1.7	0.0	0.0	0.0	0.0	0.0	8.5	1.7	0.9	35.9	23.1	15.4	87.2
**Heliconiinae Swainson, 1822**													
**Heliconiini Swainson, 1822**													
*Agraulis vanillae incarnata* (N. Riley, 1926)	5	2	1	14	10	6	13	9	2	12	7	3	84
	4.0	1.6	0.4	16.7	11.9	7.1	15.5	10.7	1.6	14.3	8.3	2.4	94.4
*Dione moneta poeyii* Butler, 1873	0	0	0	4	1	0	2	1	0	5	4	1	18
	0.0	0.0	0.0	14.8	1.9	0.0	3.7	1.9	0.0	18.5	14.8	1.9	57.4
*Dryadula phaetusa* (Linnaeus, 1758)	0	0	0	0	0	0	0	0	0	1	0	0	1
	0.0	0.0	0.0	0.0	0.0	0.0	0.0	0.0	0.0	33.3	0.0	0.0	33.3
*Dryas iulia moderata* (N. Riley, 1926)	0	0	0	13	11	3	12	9	4	12	7	1	72
	0.0	0.0	0.0	18.1	15.3	2.8	16.7	12.5	3.7	16.7	9.7	0.5	95.8
*Eueides isabella eva* (Fabricius, 1793)	0	0	0	0	0	0	0	0	0	1	0	0	1
	0.0	0.0	0.0	0.0	0.0	0.0	0.0	0.0	0.0	33.3	0.0	0.0	33.3
*Heliconius charithonia vazquezae* W. Comstock & F. Brown, 1950	7	6	1	14	11	3	14	12	5	20	10	6	109
	4.3	3.7	0.3	8.6	6.7	0.9	8.6	7.3	3.1	18.3	9.2	3.7	74.6
**Argynnini Swainson, 1833**													
*Euptoieta claudia* (Cramer, 1775)	0	0	0	7	3	0	11	8	1	5	3	1	39
	0.0	0.0	0.0	12.0	2.6	0.0	28.2	20.5	0.9	8.5	2.6	0.9	76.1
*Euptoieta hegesia meridiania* Stichel, 1938	0	2	0	4	3	1	14	14	4	14	10	2	68
	0.0	1.0	0.0	2.0	1.5	0.5	20.6	20.6	3.9	20.6	14.7	1.0	86.3
**Limenitidinae Behr, 1864**													
**Limenitidini Behr, 1864**													
*Limenitis arthemis astyanax* (Fabricius, 1775)	0	0	0	0	2	1	0	5	1	0	5	2	16
	0.0	0.0	0.0	0.0	4.2	2.1	0.0	20.8	2.1	0.0	20.8	4.2	54.2
*Adelpha eulalia* (E. Doubleday, 1848)	0	0	0	0	0	2	0	0	0	0	0	2	4
	0.0	0.0	0.0	0.0	0.0	33.3	0.0	0.0	0.0	0.0	0.0	16.7	50.0
*Adelpha paraena massilia* (C. Felder & R. Felder, 1867)	0	0	0	0	0	0	1	0	0	0	0	0	1
	0.0	0.0	0.0	0.0	0.0	0.0	33.3	0.0	0.0	0.0	0.0	0.0	33.3
*Adelpha fessonia fessonia* (Hewitson, 1847)	1	0	0	12	9	6	11	9	3	13	12	3	79
	0.4	0.0	0.0	15.2	11.4	7.6	13.9	11.4	1.3	16.5	15.2	2.5	95.4
*Adelpha basiloides* (H. Bates, 1865)	0	0	0	8	8	1	7	7	2	7	3	1	44
	0.0	0.0	0.0	18.2	18.2	0.8	10.6	15.9	1.5	10.6	2.3	0.8	78.8
**Apaturinae Boisduval, 1840**													
*Asterocampa celtis antonia* (W. H. Edwards, 1878)	0	0	0	4	0	0	9	7	0	6	8	1	35
	0.0	0.0	0.0	3.8	0.0	0.0	17.1	13.3	0.0	11.4	15.2	1.0	61.9
*Asterocampa leilia* (W. H. Edwards, 1874)	0	0	0	7	1	0	7	0	0	7	2	0	24
	0.0	0.0	0.0	19.4	1.4	0.0	19.4	0.0	0.0	19.4	2.8	0.0	62.5
*Asterocampa clyton louisa* D. Stallings & Turner, 1947	0	0	0	0	0	0	7	4	0	0	0	0	11
	0.0	0.0	0.0	0.0	0.0	0.0	42.4	24.2	0.0	0.0	0.0	0.0	66.7
*Asterocampa idyja argus* (H. Bates, 1864)	0	0	0	0	0	0	0	1	0	2	2	0	5
	0.0	0.0	0.0	0.0	0.0	0.0	0.0	6.7	0.0	13.3	26.7	0.0	46.7
*Doxocopa pavon theodora* (Lucas, 1857)	0	0	0	0	1	0	0	0	0	0	0	0	1
	0.0	0.0	0.0	0.0	33.3	0.0	0.0	0.0	0.0	0.0	0.0	0.0	33.3
*Doxocopa laure laure* (Drury, 1773)	0	0	0	0	0	0	6	1	1	4	1	0	13
	0.0	0.0	0.0	0.0	0.0	0.0	30.8	2.6	2.6	20.5	2.6	0.0	59.0
**Biblidinae Boisduval, 1833**													
**Biblidini Boisduval, 1833**													
*Biblis hyperia aganisa* Boisduval, 1836	1	1	0	13	13	6	12	10	2	9	9	1	77
	0.4	0.4	0.0	16.9	16.9	7.8	15.6	13.0	1.7	11.7	11.7	0.4	96.5
*Mestra amymone* (Ménétriés, 1857)	10	3	0	23	16	7	30	21	8	29	16	9	172
	5.8	1.2	0.0	13.4	9.3	4.1	17.4	12.2	3.1	16.9	9.3	5.2	97.9
**Catonephelini Orfila, 1952**													
*Eunica tatila tatila* (Herrich-Schäffer, 1855)	5	3	0	13	13	8	21	16	7	27	20	9	142
	1.2	0.7	0.0	9.2	9.2	5.6	14.8	11.3	3.3	19.0	14.1	6.3	94.6
*Eunica monima* (Stoll, 1782)	0	0	0	0	0	0	10	6	3	7	8	2	36
	0.0	0.0	0.0	0.0	0.0	0.0	27.8	16.7	5.6	19.4	22.2	3.7	95.4
*Myscelia ethusa ethusa* (Doyère, 1840)	0	0	0	7	5	4	12	12	6	7	9	3	65
	0.0	0.0	0.0	7.2	5.1	4.1	18.5	18.5	9.2	7.2	9.2	3.1	82.1
**Ageroniini E. Doubleday, 1847**													
*Hamadryas februa ferentina* (Godart, 1824)	4	2	0	12	8	3	8	7	5	12	13	7	81
	3.3	0.8	0.0	14.8	9.9	2.5	9.9	8.6	4.1	14.8	16.0	8.6	93.4
*Hamadryas glauconome glauconome* (H. Bates, 1864)	0	0	0	9	5	2	7	4	1	7	6	3	44
	0.0	0.0	0.0	20.5	11.4	3.0	15.9	6.1	0.8	15.9	9.1	4.5	87.1
*Hamadryas guatemalena marmarice* (Fruhstorfer, 1916)	0	0	0	0	0	0	0	0	0	2	0	0	2
	0.0	0.0	0.0	0.0	0.0	0.0	0.0	0.0	0.0	33.3	0.0	0.0	33.3
**Epiphelini Jenkins, 1987**													
*Epiphile adrasta adrasta* Hewitson, 1861	0	0	0	0	3	3	1	7	3	1	8	6	32
	0.0	0.0	0.0	0.0	3.1	3.1	1.0	14.6	6.3	1.0	25.0	18.8	72.9
**Eubagini Burmeister, 1878**													
*Dynamine dyonis* Geyer, 1837	0	0	0	0	0	0	3	1	0	3	1	0	8
	0.0	0.0	0.0	0.0	0.0	0.0	25.0	4.2	0.0	12.5	4.2	0.0	45.8
**Cyrestinae Guenée, 1865**													
**Cyrestini Guenée, 1865**													
*Marpesia chiron* (Fabricius, 1775)	0	0	0	0	0	0	0	0	0	1	0	0	1
	0.0	0.0	0.0	0.0	0.0	0.0	0.0	0.0	0.0	33.3	0.0	0.0	33.3
**Marpesia petreus* (Cramer, 1776)	0	0	0	0	0	0	1	0	0	4	0	0	5
	0.0	0.0	0.0	0.0	0.0	0.0	6.7	0.0	0.0	80.0	0.0	0.0	86.7
**Nymphalinae Rafinesque, 1815**													
**Coeini Scudder, 1893**													
*Historis acheronta acheronta* (Fabricius, 1775)	0	0	0	0	0	0	0	0	0	0	1	0	1
	0.0	0.0	0.0	0.0	0.0	0.0	0.0	0.0	0.0	0.0	33.3	0.0	33.3
**Nymphalini Rafinesque, 1815**													
**Smyrna blomfildia datis* Fruhstorfer, 1908	0	0	0	0	3	1	3	1	0	0	9	1	18
	0.0	0.0	0.0	0.0	11.1	1.9	5.6	1.9	0.0	0.0	50.0	1.9	72.2
*Vanessa virginiensis* (Drury, 1773)	0	0	0	2	1	1	6	5	2	3	1	0	21
	0.0	0.0	0.0	3.2	1.6	1.6	19.0	15.9	6.3	4.8	1.6	0.0	54.0
*Vanessa cardui* (Linnaeus, 1758)	0	0	0	0	4	0	2	4	0	6	3	0	19
	0.0	0.0	0.0	0.0	14.0	0.0	3.5	14.0	0.0	21.1	10.5	0.0	63.2
*Vanessa atalanta rubria* (Fruhstorfer, 1909)	0	0	0	14	10	8	12	16	10	22	10	8	110
	0.0	0.0	0.0	12.7	9.1	7.3	10.9	14.5	9.1	20.0	9.1	7.3	100
*Nymphalis antiopa antiopa* (Linnaeus, 1758)	0	0	0	0	0	0	0	0	0	0	1	0	1
	0.0	0.0	0.0	0.0	0.0	0.0	0.0	0.0	0.0	0.0	33.3	0.0	33.3
*Polygonia interrogationis* (Fabricius, 1798)	0	0	0	0	3	0	2	4	0	0	2	0	11
	0.0	0.0	0.0	0.0	9.1	0.0	6.1	12.1	0.0	0.0	6.1	0.0	33.3
**Victorinini Scudder, 1893**													
*Anartia jatrophae luteipicta* (Fruhstorfer, 1907)	3	2	0	5	2	2	14	13	6	22	11	5	85
	1.2	0.8	0.0	2.0	0.8	0.8	16.5	15.3	7.1	25.9	12.9	3.9	87.1
**Anartia fatima fatima* (Fabricius, 1793)	0	0	0	21	3	0	8	7	0	18	1	0	58
	0.0	0.0	0.0	36.2	1.7	0.0	9.2	8.0	0.0	31.0	0.6	0.0	86.8
*Siproeta stelenes biplagiata* (Fruhstorfer, 1907)	0	0	0	3	3	1	8	6	3	6	5	0	35
	0.0	0.0	0.0	2.9	2.9	1.0	15.2	11.4	5.7	11.4	9.5	0.0	60.0
**Junoniini Reuter, 1896**													
*Junonia coenia coenia* Hübner, 1822	0	0	0	2	1	0	0	0	0	4	1	0	8
	0.0	0.0	0.0	8.3	4.2	0.0	0.0	0.0	0.0	33.3	4.2	0.0	50.0
*Junonia evarete* (Cramer, 1779)	0	0	0	0	0	0	0	0	0	0	1	0	1
	0.0	0.0	0.0	0.0	0.0	0.0	0.0	0.0	0.0	0.0	33.3	0.0	33.3
**Melitaeini Newman, 1870**													
*Chlosyne janais janais* (Drury, 1782)	5	0	0	13	9	4	16	6	1	11	12	2	79
	4.2	0.0	0.0	16.5	11.4	3.4	20.3	7.6	0.4	13.9	15.2	1.7	94.5
*Chlosyne definita definita* (E. Aaron, 1885)	0	0	0	3	0	0	2	0	0	1	0	0	6
	0.0	0.0	0.0	33.3	0.0	0.0	11.1	0.0	0.0	5.6	0.0	0.0	50.0
*Chlosyne melitaeoides* (C. Felder & R. Felder, 1867)	0	0	0	0	0	0	3	1	0	9	0	0	13
	0.0	0.0	0.0	0.0	0.0	0.0	7.7	2.6	0.0	46.2	0.0	0.0	56.4
*Chlosyne endeis pardelina* Scott, 1986	0	0	0	0	0	0	2	0	0	5	1	0	8
	0.0	0.0	0.0	0.0	0.0	0.0	8.3	0.0	0.0	41.7	4.2	0.0	54.2
*Chlosyne rosita browni* Bauer, 1961	0	0	0	0	0	0	12	8	1	14	8	5	48
	0.0	0.0	0.0	0.0	0.0	0.0	16.7	11.1	0.7	19.4	11.1	6.9	66.0
**Chlosyne theona bollii* (W. H. Edwards, 1877)	0	0	0	0	0	0	8	0	0	3	0	0	11
	0.0	0.0	0.0	0.0	0.0	0.0	72.7	0.0	0.0	18.2	0.0	0.0	90.9
*Chlosyne lacinia adjutrix* Scudder, 1875	2	0	0	7	4	1	11	8	0	19	7	0	59
	1.1	0.0	0.0	7.9	4.5	0.6	12.4	9.0	0.0	32.2	7.9	0.0	75.7
**Microtia elva elva* H. Bates, 1864	0	0	0	0	0	0	14	1	0	35	2	0	52
	0.0	0.0	0.0	0.0	0.0	0.0	26.9	0.6	0.0	67.3	1.3	0.0	96.2
*Texola elada ulrica* (W. H. Edwards, 1877)	0	0	0	0	0	0	1	0	0	2	0	0	3
	0.0	0.0	0.0	0.0	0.0	0.0	11.1	0.0	0.0	22.2	0.0	0.0	33.3
*Anthanassa texana texana* (W. H. Edwards, 1863)	2	3	0	18	14	6	10	15	7	15	11	1	102
	0.7	1.0	0.0	17.6	13.7	5.9	9.8	14.7	4.6	14.7	10.8	0.3	93.8
*Anthanassa ardys* (Hewitson, 1864)	0	0	0	0	3	1	0	0	0	0	1	0	5
	0.0	0.0	0.0	0.0	40.0	6.7	0.0	0.0	0.0	0.0	6.7	0.0	53.3
*Anthanassa ptolyca* (H. Bates, 1864)	0	0	0	0	0	0	0	1	0	0	0	0	1
	0.0	0.0	0.0	0.0	0.0	0.0	0.0	33.3	0.0	0.0	0.0	0.0	33.3
*Anthanassa argentea* (Godman & Salvin, 1882)	1	1	0	10	13	3	10	9	2	12	14	1	76
	0.4	0.4	0.0	13.2	17.1	2.6	13.2	11.8	0.9	15.8	18.4	0.4	94.3
*Anthanassa tulcis* (H. Bates, 1864)	0	0	0	0	0	1	0	0	3	0	2	2	8
	0.0	0.0	0.0	0.0	0.0	4.2	0.0	0.0	25.0	0.0	8.3	8.3	45.8
*Phyciodes graphica* (R. Felder, 1869)	3	0	0	8	5	0	8	7	0	8	7	0	46
	2.2	0.0	0.0	17.4	10.9	0.0	17.4	10.1	0.0	17.4	10.1	0.0	85.5
*Phyciodes mylitta mexicana* A. Hall, 1928	0	0	0	1	0	0	4	0	0	3	0	0	8
	0.0	0.0	0.0	4.2	0.0	0.0	33.3	0.0	0.0	12.5	0.0	0.0	50.0
*Phyciodes phaon phaon* (W. H. Edwards, 1864)	0	0	0	4	0	0	0	0	0	1	0	0	5
	0.0	0.0	0.0	53.3	0.0	0.0	0.0	0.0	0.0	6.7	0.0	0.0	60.0
*Phyciodes tharos tharos* (Drury, 1773)	6	2	0	8	14	4	7	14	1	14	10	6	86
	7.0	0.8	0.0	9.3	16.3	4.7	8.1	16.3	0.4	16.3	11.6	7.0	97.7
**Charaxinae Guenée, 1865**													
**Anaeini Reuter, 1896**													
*Anaea aidea* (Guérin-Méneville, 1844)	16	6	4	40	28	17	79	58	28	77	50	39	442
	3.6	0.9	0.6	9.0	6.3	3.8	17.9	13.1	6.3	17.4	11.3	8.8	99.2
*Anaea andria* Scudder, 1875	0	0	0	9	5	5	10	8	3	17	10	6	73
	0.0	0.0	0.0	12.3	4.6	4.6	13.7	11.0	1.4	23.3	13.7	8.2	92.7
*Fountainea glycerium glycerium* (E. Doubleday, 1849)	0	0	0	6	5	2	5	3	0	0	2	0	23
	0.0	0.0	0.0	17.4	21.7	2.9	21.7	8.7	0.0	0.0	2.9	0.0	75.4
*Memphis pithyusa pithyusa* (R. Felder, 1869)	0	0	0	1	0	0	2	1	0	4	3	0	11
	0.0	0.0	0.0	3.0	0.0	0.0	6.1	3.0	0.0	24.2	9.1	0.0	45.5
**Satyrinae Boisduval, 1833**													
**Satyrini Boisduval, 1833**													
*Cyllopsis* sp. R. Felder, 1869	0	0	0	0	0	4	0	3	3	0	0	3	13
	0.0	0.0	0.0	0.0	0.0	20.5	0.0	7.7	15.4	0.0	0.0	7.7	51.3
*Cyllopsis dospassosi* L. Miller, 1974	1	1	0	9	3	0	11	8	1	8	5	1	48
	0.7	0.7	0.0	18.8	4.2	0.0	22.9	16.7	0.7	16.7	6.9	0.7	88.9
*Cyllopsis gemma freemani* (D. Stallings & Turner, 1947)	6	2	1	23	15	7	19	14	7	20	14	7	135
	4.4	1.0	0.2	17.0	11.1	5.2	14.1	10.4	5.2	14.8	10.4	3.5	97.3
*Megisto rubricata rubricata* (W. H. Edwards, 1871)	0	0	0	4	1	0	6	0	0	2	0	0	13
	0.0	0.0	0.0	20.5	2.6	0.0	30.8	0.0	0.0	5.1	0.0	0.0	59.0
*Hermeuptychia hermes* (Fabricius, 1775)	0	0	0	14	7	2	12	6	1	12	6	1	61
	0.0	0.0	0.0	23.0	7.7	1.1	19.7	6.6	0.5	19.7	6.6	0.5	85.2
**Hesperiidae Latreille, 1809**													
**Eudaminae Mabille, 1877**													
*Phocides polybius lilea* (Reakirt, 1867)	0	0	0	0	0	0	2	0	0	0	0	0	2
	0.0	0.0	0.0	0.0	0.0	0.0	33.3	0.0	0.0	0.0	0.0	0.0	33.3
*Phocides urania urania* (Westwood, 1852)	0	0	0	0	0	0	3	0	0	0	2	0	5
	0.0	0.0	0.0	0.0	0.0	0.0	20.0	0.0	0.0	0.0	13.3	0.0	33.3
*Epargyreus socus orizaba* Scudder, 1872	0	0	0	3	1	0	2	0	0	2	0	0	8
	0.0	0.0	0.0	12.5	4.2	0.0	8.3	0.0	0.0	8.3	0.0	0.0	33.3
*Polygonus leo arizonensis* (Skinner, 1911)	0	0	0	0	2	0	0	0	0	0	0	0	2
	0.0	0.0	0.0	0.0	33.3	0.0	0.0	0.0	0.0	0.0	0.0	0.0	33.3
*Chioides albofasciatus* (Hewitson, 1867)	0	2	0	8	12	3	7	3	0	10	6	1	52
	0.0	1.3	0.0	15.4	23.1	3.8	9.0	3.8	0.0	19.2	11.5	0.6	87.8
**Chioides zilpa* (Butler, 1872)	0	0	0	11	7	2	6	0	0	9	6	1	42
	0.0	0.0	0.0	26.2	16.7	1.6	9.5	0.0	0.0	21.4	14.3	0.8	90.5
*Aguna asander asander* (Hewitson, 1867)	0	0	0	1	0	0	4	0	0	0	0	0	5
	0.0	0.0	0.0	6.7	0.0	0.0	53.3	0.0	0.0	0.0	0.0	0.0	60.0
*Aguna metophis* (Latreille, 1824)	0	0	0	0	0	0	1	0	0	1	0	0	2
	0.0	0.0	0.0	0.0	0.0	0.0	16.7	0.0	0.0	16.7	0.0	0.0	33.3
*Typhedanus undulatus* (Hewitson, 1867)	0	0	0	0	0	0	3	0	0	5	0	0	8
	0.0	0.0	0.0	0.0	0.0	0.0	12.5	0.0	0.0	41.7	0.0	0.0	54.2
*Codatractus bryaxis* (Hewitson, 1867)	0	0	0	1	0	0	0	0	0	0	0	0	1
	0.0	0.0	0.0	33.3	0.0	0.0	0.0	0.0	0.0	0.0	0.0	0.0	33.3
*Urbanus proteus proteus* (Linnaeus, 1758)	0	0	0	2	0	0	0	0	0	0	0	0	2
	0.0	0.0	0.0	33.3	0.0	0.0	0.0	0.0	0.0	0.0	0.0	0.0	33.3
*Urbanus dorantes dorantes* (Stoll, 1790)	0	0	0	1	0	0	1	0	0	0	1	0	3
	0.0	0.0	0.0	11.1	0.0	0.0	11.1	0.0	0.0	0.0	11.1	0.0	33.3
**Urbanus procne* (Plötz, 1881)	0	0	0	5	0	0	2	1	0	3	1	0	12
	0.0	0.0	0.0	41.7	0.0	0.0	5.6	2.8	0.0	16.7	2.8	0.0	69.4
*Urbanus teleus* (Hübner, 1821)	0	0	0	4	0	0	1	0	0	3	0	0	8
	0.0	0.0	0.0	33.3	0.0	0.0	4.2	0.0	0.0	12.5	0.0	0.0	50.0
*Urbanus doryssus* (Swainson, 1831)	0	0	0	5	1	0	4	1	1	5	2	0	19
	0.0	0.0	0.0	17.5	1.8	0.0	7.0	1.8	1.8	26.3	7.0	0.0	63.2
*Astraptes fulgerator azul* (Reakirt, 1867)	1	0	0	7	3	2	7	2	0	7	2	0	31
	1.1	0.0	0.0	15.1	6.5	2.2	15.1	2.2	0.0	15.1	2.2	0.0	59.1
*Astraptes alector hopfferi* (Plötz, 1881)	0	0	0	0	0	0	0	0	2	0	0	0	2
	0.0	0.0	0.0	0.0	0.0	0.0	0.0	0.0	33.3	0.0	0.0	0.0	33.3
*Autochton cellus* (Boisduval & Le Conte, 1837)	0	0	0	0	1	4	0	1	5	0	2	0	13
	0.0	0.0	0.0	0.0	2.6	10.3	0.0	2.6	25.6	0.0	5.1	0.0	46.2
*Autochton cincta* (Plötz, 1882)	0	1	0	0	0	6	0	5	4	0	3	2	21
	0.0	1.6	0.0	0.0	0.0	19.0	0.0	15.9	6.3	0.0	9.5	3.2	55.6
*Achalarus toxeus* (Plötz, 1882)	0	0	0	8	3	0	6	1	0	6	1	0	25
	0.0	0.0	0.0	32.0	8.0	0.0	16.0	1.3	0.0	16.0	1.3	0.0	74.7
*Thorybes pylades albosuffusa* H. Freeman, 1943	0	0	0	5	3	2	0	0	0	0	1	0	11
	0.0	0.0	0.0	30.3	18.2	6.1	0.0	0.0	0.0	0.0	3.0	0.0	57.6
*Cabares potrillo potrillo* (Lucas, 1857)	0	1	0	7	9	1	0	4	0	9	6	1	38
	0.0	0.9	0.0	18.4	23.7	0.9	0.0	3.5	0.0	15.8	10.5	0.9	74.6
*Spathilepia clonius* (Cramer, 1775)	0	0	0	0	2	0	0	0	0	2	0	0	4
	0.0	0.0	0.0	0.0	16.7	0.0	0.0	0.0	0.0	33.3	0.0	0.0	50.0
*Cogia hippalus hiska* Evans, 1953	0	0	0	0	0	0	0	0	0	0	0	2	2
	0.0	0.0	0.0	0.0	0.0	0.0	0.0	0.0	0.0	0.0	0.0	33.3	33.3
**Pyrginae Burmeister, 1878**													
**Carcharodini Verity, 1940**													
*Arteurotia tractipennis tractipennis* Butler & H. Druce, 1872	0	0	0	0	2	0	1	0	0	0	1	0	4
	0.0	0.0	0.0	0.0	16.7	0.0	8.3	0.0	0.0	0.0	8.3	0.0	33.3
*Polyctor enops* (Godman & Salvin, 1894)	0	0	0	0	0	0	0	4	0	0	2	0	6
	0.0	0.0	0.0	0.0	0.0	0.0	0.0	44.4	0.0	0.0	11.1	0.0	55.6
*Noctuana lactifera bipuncta* (Plötz, 1884)	0	0	0	0	0	0	3	3	0	0	3	0	9
	0.0	0.0	0.0	0.0	0.0	0.0	11.1	22.2	0.0	0.0	22.2	0.0	55.6
*Bolla brennus brennus* (Godman & Salvin, 1896)	0	0	0	1	0	0	0	0	0	0	0	0	1
	0.0	0.0	0.0	33.3	0.0	0.0	0.0	0.0	0.0	0.0	0.0	0.0	33.3
*Bolla clytius* (Godman & Salvin, 1897)	0	0	0	0	0	0	0	0	1	0	0	1	2
	0.0	0.0	0.0	0.0	0.0	0.0	0.0	0.0	16.7	0.0	0.0	16.7	33.3
*Staphylus mazans* (Reakirt, 1867)	0	1	0	9	6	2	4	3	1	6	3	0	35
	0.0	1.0	0.0	25.7	17.1	3.8	7.6	5.7	1.0	11.4	2.9	0.0	76.2
*Staphylus azteca* (Scudder, 1872)	0	0	0	0	1	0	0	3	0	0	0	0	4
	0.0	0.0	0.0	0.0	8.3	0.0	0.0	50.0	0.0	0.0	0.0	0.0	58.3
*Pholisora catullus* (Fabricius, 1793)	0	0	0	1	0	0	3	0	0	2	0	0	6
	0.0	0.0	0.0	5.6	0.0	0.0	16.7	0.0	0.0	11.1	0.0	0.0	33.3
**Erynnini Brues & F. Carpenter, 1932**													
*Gorgythion begga pyralina* (Möschler, 1877)	2	4	1	2	3	0	0	0	0	0	0	0	12
	5.6	11.1	2.8	5.6	8.3	0.0	0.0	0.0	0.0	0.0	0.0	0.0	33.3
*Grais stigmaticus stigmaticus* (Mabille, 1883)	0	0	0	0	3	0	0	3	0	0	5	0	11
	0.0	0.0	0.0	0.0	9.1	0.0	0.0	18.2	0.0	0.0	30.3	0.0	57.6
*Timochares ruptifasciata* (Plötz, 1884)	0	0	0	1	0	0	4	1	0	4	1	0	11
	0.0	0.0	0.0	3.0	0.0	0.0	24.2	3.0	0.0	24.2	3.0	0.0	57.6
*Chiomara georgina georgina* (Reakirt, 1868)	5	4	1	22	14	5	12	13	5	19	10	3	113
	2.9	2.4	0.3	19.5	12.4	2.9	10.6	11.5	2.9	16.8	8.8	1.8	92.9
*Gesta invisus* (Butler & H. Druce, 1872)	0	0	0	9	4	3	6	4	0	9	11	3	49
	0.0	0.0	0.0	12.2	5.4	4.1	4.1	2.7	0.0	18.4	22.4	4.1	73.5
*Erynnis tristis tatius* (W. H. Edwards, 1883)	0	1	0	7	7	2	8	7	2	12	11	5	62
	0.0	0.5	0.0	7.5	7.5	2.2	8.6	7.5	2.2	19.4	17.7	5.4	78.5
**Erynnis funeralis* (Scudder & Burgess, 1870)	1	0	0	5	0	0	0	0	0	6	0	0	12
	2.8	0.0	0.0	41.7	0.0	0.0	0.0	0.0	0.0	33.3	0.0	0.0	77.8
**Achlyodidini Burmeister, 1878**													
**Achlyodes pallida* (R. Felder, 1869)	0	0	0	0	3	0	0	2	0	0	6	0	11
	0.0	0.0	0.0	0.0	9.1	0.0	0.0	6.1	0.0	0.0	54.5	0.0	69.7
*Eantis tamenund* (W. H. Edwards, 1871)	6	4	0	16	11	5	14	10	3	19	14	6	108
	5.6	2.5	0.0	14.8	10.2	4.6	13.0	9.3	1.9	17.6	13.0	5.6	97.8
*Zera hyacinthinius hyacinthinus* (Mabille, 1877)	0	0	0	0	2	0	0	2	0	1	0	0	5
	0.0	0.0	0.0	0.0	13.3	0.0	0.0	26.7	0.0	6.7	0.0	0.0	46.7
**Pyrgini Burmeister, 1878**													
*Carrhenes canescens canescens* (R. Felder, 1869)	1	0	0	0	0	0	0	0	0	1	0	0	2
	16.7	0.0	0.0	0.0	0.0	0.0	0.0	0.0	0.0	16.7	0.0	0.0	33.3
*Systasea pulverulenta* (R. Felder, 1869)	0	0	0	2	2	0	1	1	0	2	0	0	8
	0.0	0.0	0.0	8.3	16.7	0.0	4.2	4.2	0.0	16.7	0.0	0.0	50.0
*Celotes nessus* (W. H. Edwards, 1877)	0	0	0	0	2	0	0	0	0	2	1	0	5
	0.0	0.0	0.0	0.0	26.7	0.0	0.0	0.0	0.0	13.3	6.7	0.0	46.7
*Pyrgus communis communis* (Grote, 1872)	0	0	0	2	1	1	14	12	7	3	4	1	45
	0.0	0.0	0.0	1.5	0.7	0.7	31.1	26.7	15.6	2.2	3.0	0.7	82.2
*Pyrgus albescens* Plötz, 1884	2	4	0	13	13	7	12	10	4	21	10	7	103
	0.6	2.6	0.0	12.6	12.6	6.8	11.7	9.7	2.6	20.4	9.7	6.8	96.1
*Pyrgus oileus* (Linnaeus, 1767)	6	7	1	23	18	9	22	24	12	26	17	11	176
	2.3	4.0	0.2	13.1	10.2	5.1	12.5	13.6	6.8	14.8	9.7	6.3	98.5
*Pyrgus philetas* W. H. Edwards, 1881	0	2	0	0	3	1	0	0	0	0	5	1	12
	0.0	11.1	0.0	0.0	8.3	2.8	0.0	0.0	0.0	0.0	27.8	2.8	52.8
*Heliopyrgus domicella domicella* (Erichson, 1849)	1	0	0	1	0	0	0	0	0	0	0	0	2
	16.7	0.0	0.0	16.7	0.0	0.0	0.0	0.0	0.0	0.0	0.0	0.0	33.3
*Heliopyrgus sublinea* (Schaus, 1902)	0	0	0	0	0	0	3	2	0	0	1	0	6
	0.0	0.0	0.0	0.0	0.0	0.0	16.7	22.2	0.0	0.0	5.6	0.0	44.4
*Heliopetes laviana laviana* (Hewitson, 1868)	5	2	0	11	10	4	10	12	1	23	17	6	101
	3.3	0.7	0.0	10.9	9.9	4.0	9.9	11.9	0.3	22.8	16.8	5.9	96.4
*Heliopetes macaira macaira* (Reakirt, 1867)	0	0	0	1	0	0	0	0	0	0	0	0	1
	0.0	0.0	0.0	33.3	0.0	0.0	0.0	0.0	0.0	0.0	0.0	0.0	33.3
**Hesperiinae Latreille, 1809**													
**Megathymini J. H. Comstock & A. Comstock, 1895**													
*Agathymus remingtoni* (D. Stallings & Turner, 1958)	0	0	0	0	0	0	1	0	0	0	0	0	1
	0.0	0.0	0.0	0.0	0.0	0.0	33.3	0.0	0.0	0.0	0.0	0.0	33.3
**Thymelicini Tutt, 1905**													
*Ancyloxypha arene* (W. H. Edwards, 1871)	0	0	0	0	0	4	0	0	1	0	0	3	8
	0.0	0.0	0.0	0.0	0.0	33.3	0.0	0.0	4.2	0.0	0.0	12.5	50.0
**Oarisma edwardsii* (W. Barnes, 1897)	0	0	0	0	0	1	0	0	9	0	0	2	12
	0.0	0.0	0.0	0.0	0.0	2.8	0.0	0.0	75.0	0.0	0.0	5.6	83.3
*Copaeodes aurantiaca* (Hewitson, 1868)	0	0	0	0	0	2	0	0	0	0	0	1	3
	0.0	0.0	0.0	0.0	0.0	44.4	0.0	0.0	0.0	0.0	0.0	11.1	55.6
*Copaeodes minima* (W. H. Edwards, 1870)	0	0	0	0	0	0	0	0	1	0	0	1	2
	0.0	0.0	0.0	0.0	0.0	0.0	0.0	0.0	16.7	0.0	0.0	16.7	33.3
**Calpodini A. Clark, 1948**													
*Panoquina lucas* (Fabricius, 1793)	0	0	0	0	0	0	0	1	0	0	0	0	1
	0.0	0.0	0.0	0.0	0.0	0.0	0.0	33.3	0.0	0.0	0.0	0.0	33.3
**Anthoptini A. Warren, 2009**													
*Synapte pecta* Evans, 1955	0	0	0	0	0	0	0	1	0	0	2	0	3
	0.0	0.0	0.0	0.0	0.0	0.0	0.0	11.1	0.0	0.0	22.2	0.0	33.3
**Moncini A. Warren, 2008**													
*Mnasicles geta* Godman, 1901	0	0	0	0	0	0	0	2	0	0	0	0	2
	0.0	0.0	0.0	0.0	0.0	0.0	0.0	33.3	0.0	0.0	0.0	0.0	33.3
*Remella rita* (Evans, 1955)	0	0	0	0	0	0	0	1	3	0	2	2	8
	0.0	0.0	0.0	0.0	0.0	0.0	0.0	4.2	25.0	0.0	8.3	16.7	54.2
*Amblyscirtes aenus erna* H. Freeman, 1943	0	0	0	0	1	0	0	0	2	0	0	1	4
	0.0	0.0	0.0	0.0	8.3	0.0	0.0	0.0	16.7	0.0	0.0	8.3	33.3
**Amblyscirtes celia* Skinner, 1895	0	0	0	0	0	2	0	1	6	0	2	0	11
	0.0	0.0	0.0	0.0	0.0	6.1	0.0	3.0	54.5	0.0	6.1	0.0	69.7
*Amblyscirtes fimbriata fimbriata* (Plötz, 1882)	0	0	0	0	0	0	2	1	0	0	0	0	3
	0.0	0.0	0.0	0.0	0.0	0.0	22.2	11.1	0.0	0.0	0.0	0.0	33.3
*Amblyscirtes anubis* (Godman, 1900)	0	0	0	1	0	0	2	2	0	0	0	0	5
	0.0	0.0	0.0	6.7	0.0	0.0	13.3	26.7	0.0	0.0	0.0	0.0	46.7
*Repens florus* (Godman, 1900)	0	0	0	5	5	1	0	0	0	5	2	0	18
	0.0	0.0	0.0	9.3	18.5	1.9	0.0	0.0	0.0	18.5	3.7	0.0	51.9
*Monca crispinus* (Plötz, 1882)	1	0	0	0	0	0	0	0	0	2	0	0	3
	11.1	0.0	0.0	0.0	0.0	0.0	0.0	0.0	0.0	22.2	0.0	0.0	33.3
*Nastra julia* (H. Freeman, 1945)	0	0	0	0	0	1	0	0	0	0	0	0	1
	0.0	0.0	0.0	0.0	0.0	33.3	0.0	0.0	0.0	0.0	0.0	0.0	33.3
*Cymaenes trebius* (Mabille, 1891)	0	0	0	0	3	1	0	0	0	0	0	1	5
	0.0	0.0	0.0	0.0	40.0	6.7	0.0	0.0	0.0	0.0	0.0	6.7	53.3
*Lerodea eufala eufala* (W. H. Edwards, 1869)	0	0	0	0	4	0	2	0	0	0	0	0	6
	0.0	0.0	0.0	0.0	44.4	0.0	11.1	0.0	0.0	0.0	0.0	0.0	55.6
*Lerodea arabus* (W. H. Edwards, 1882)	0	0	0	1	0	0	1	0	0	0	0	0	2
	0.0	0.0	0.0	16.7	0.0	0.0	16.7	0.0	0.0	0.0	0.0	0.0	33.3
*Lerema accius* (J. E. Smith, 1797)	3	1	0	11	7	2	5	0	0	1	2	0	32
	3.1	1.0	0.0	22.9	14.6	2.1	5.2	0.0	0.0	1.0	2.1	0.0	52.1
*Lerema liris* Evans, 1955	0	0	0	0	0	0	8	8	3	2	3	2	26
	0.0	0.0	0.0	0.0	0.0	0.0	10.3	20.5	7.7	2.6	3.8	2.6	47.4
*Vettius fantasos* (Cramer, 1780)	0	0	0	1	0	0	2	0	0	0	0	0	3
	0.0	0.0	0.0	11.1	0.0	0.0	22.2	0.0	0.0	0.0	0.0	0.0	33.3
**Hesperiini Latreille, 1809**													
*Hylephila phyleus phyleus* (Drury, 1773)	0	0	0	5	2	0	5	0	0	0	0	0	12
	0.0	0.0	0.0	13.9	5.6	0.0	27.8	0.0	0.0	0.0	0.0	0.0	47.2
*Polites vibex praeceps* (Scudder, 1872)	0	0	0	2	0	0	3	0	0	0	0	0	5
	0.0	0.0	0.0	13.3	0.0	0.0	20.0	0.0	0.0	0.0	0.0	0.0	33.3
*Wallengrenia otho otho* (J. E. Smith, 1797)	0	0	0	0	0	0	0	0	0	1	0	0	1
	0.0	0.0	0.0	0.0	0.0	0.0	0.0	0.0	0.0	33.3	0.0	0.0	33.3
*Atalopedes campestris huron* (W. H. Edwards, 1863)	0	0	0	0	3	2	0	0	5	0	2	6	18
	0.0	0.0	0.0	0.0	5.6	3.7	0.0	0.0	9.3	0.0	3.7	22.2	44.4
*Poanes zabulon* (Boisduval & Le Conte, 1837)	0	0	0	0	0	1	0	0	0	0	2	0	3
	0.0	0.0	0.0	0.0	0.0	11.1	0.0	0.0	0.0	0.0	22.2	0.0	33.3
*Poanes melane vitellina* (Herrich-Schäffer, 1869)	0	0	0	0	0	1	0	0	0	0	0	1	2
	0.0	0.0	0.0	0.0	0.0	16.7	0.0	0.0	0.0	0.0	0.0	16.7	33.3
*Quasimellana eulogius* (Plötz, 1882)	0	0	0	0	0	0	0	0	0	0	1	0	1
	0.0	0.0	0.0	0.0	0.0	0.0	0.0	0.0	0.0	0.0	33.3	0.0	33.3
**Quinta cannae* (Herrich-Schäffer, 1869)	0	0	0	0	0	4	0	0	0	0	1	0	5
	0.0	0.0	0.0	0.0	0.0	80.0	0.0	0.0	0.0	0.0	6.7	0.0	86.7
*Nyctelius nyctelius nyctelius* (Latreille, 1824)	0	0	0	2	0	0	3	5	1	0	0	2	13
	0.0	0.0	0.0	5.1	0.0	0.0	7.7	12.8	2.6	0.0	0.0	5.1	33.3
